# Pulmonary effects of exposure to indium and its compounds: cross-sectional survey of exposed workers and experimental findings in rodents

**DOI:** 10.1186/s12989-022-00510-w

**Published:** 2022-12-20

**Authors:** Nan Liu, Yi Guan, Yan Yu, Gai Li, Ling Xue, Weikang Li, Xiaoyu Qu, Ning Li, Sanqiao Yao

**Affiliations:** 1 School of Public Health, North China University of Science and Technology, Key Laboratory of Coal Mine Health and Safety in Hebei, Tangshan, 063210 Hebei China; 2 North China University of Science and Technology Affiliated Hospital, Tangshan, 063000 Hebei China; 3 Xinxiang Medical University, Xinxiang, 453003 Henan China

**Keywords:** Biomarkers, Occupational exposure, Indium tin oxide, Indium oxide, Indium sulfate, Indium trichloride

## Abstract

**Background:**

Many studies have shown that occupational exposure to indium and its compounds could induce lung disease. Although animal toxicological studies and human epidemiological studies suggest indium exposure may cause lung injury, inflammation, pulmonary fibrosis, emphysema, pulmonary alveolar proteinosis, and even lung cancer, related data collected from humans is currently limited and confined to single workplaces, and the early effects of exposure on the lungs are not well understood.

**Objectives:**

This study combined population studies and animal experiments to examine the links of indium with pulmonary injury, as well as its mechanism of action. A cross-sectional epidemiological study of indium-exposed workers from China was conducted to evaluate associations between occupational indium exposure and serum biomarkers of early effect. This study also compares and analyzes the causal perspectives of changes in human serum biomarkers induced by indium compound exposure and indium exposure-related rat lung pathobiology, and discusses possible avenues for their recognition and prevention.

**Methods:**

This is a study of 57 exposed (at least 6 h per day for one year) workers from an indium ingot production plant, and 63 controls. Indium concentration in serum, urine, and airborne as exposure indices were measured by inductively coupled plasma-mass spectrometry. Sixteen serum biomarkers of pulmonary injury, inflammation, and oxidative stress were measured using ELISA. The associations between serum indium and 16 serum biomarkers were analyzed to explore the mechanism of action of indium on pulmonary injury in indium-exposed workers. Animal experiments were conducted to measure inflammatory factors levels in bronchoalveolar lavage fluid (BALF) and lung tissue protein expressions in rats. Four different forms of indium compound-exposed rat models were established (intratracheal instillation twice per week, 8 week exposure, 8 week recovery). Model I: 0, 1.2, 3, and 6 mg/kg bw indium tin oxide group; Model II: 0, 1.2, 3, and 6 mg/kg bw indium oxide (In_2_O_3_) group; Model III: 0, 0.523, 1.046, and 2.614 mg/kg bw indium sulfate (In_2_(SO_4_)_3_) group; Model IV: 0, 0.065, 0.65, and 1.3 mg/kg bw indium trichloride (InCl_3_) group. Lung pathological changes were assessed by hematoxylin & eosin, periodic acid Schiff, and Masson’s staining, transmission electron microscopy, and the protein changes were determined by immunohistochemistry.

**Results:**

In the production workshop, the airborne indium concentration was 78.4 μg/m^3^. The levels of serum indium and urine indium in indium-exposed workers were 39.3 μg/L and 11.0 ng/g creatinine. Increased lung damage markers, oxidative stress markers, and inflammation markers were found in indium-exposed workers. Serum indium levels were statistically and positively associated with the serum levels of SP-A, IL-1β, IL-6 in indium-exposed workers. Among them, SP-A showed a duration-response pattern. The results of animal experiments showed that, with an increase in dosage, indium exposure significantly increased the levels of serum indium and lung indium, as well as the BALF levels of IL‑1β, IL‑6, IL‑10, and TNF‑α and up-regulated the protein expression of SP-A, SP-D, KL-6, GM-CSF, NF-κB p65, and HO-1 in all rat models groups. TEM revealed that In_2_(SO_4_)_3_ and InCl_3_ are soluble and that no particles were found in lung tissue, in contrast to the non-soluble compounds (ITO and In_2_O_3_). No PAS-staining positive substance was found in the lung tissue of In_2_(SO_4_)_3_ and InCl_3_ exposure groups, whereas ITO and In_2_O_3_ rat models supported findings of pulmonary alveolar proteinosis and interstitial fibrosis seen in human indium lung disease. ITO and InCl_3_ can accelerate interstitial fibrosis. Findings from our in vivo studies demonstrated that intra-alveolar accumulation of surfactant (immunohistochemistry) and characteristic cholesterol clefts granulomas of indium lung disease (PAS staining) were triggered by a specific form of indium (ITO and In_2_O_3_).

**Conclusions:**

In indium-exposed workers, biomarker findings indicated lung damage, oxidative stress and an inflammatory response. In rat models of the four forms of indium encountered in a workplace, the biomarkers response to all compounds overall corresponded to that in humans. In addition, pulmonary alveolar proteinosis was found following exposure to indium tin oxide and indium oxide in the rat models, and interstitial fibrosis was found following exposure to indium tin oxide and indium trichloride, supporting previous report of human disease. Serum SP-A levels were positively associated with indium exposure and may be considered a potential biomarker of exposure and effect in exposed workers.

## Introduction

Indium lung disease, a new and potentially fatal occupational disease, is characterized by pulmonary alveolar proteinosis that may progress to fibrosis, with or without emphysema. The disease was first reported in 2003 [1]. The major demand for indium has been in the form of indium tin oxide (ITO), targeted for the use in the transparent conductive coating of liquid–crystal displays (LCD). Since 2003, 20 cases of advanced lung disease were reported in young workers exposed to indium compounds during the production, use, or reclamation of ITO [1–6]. The disease continues to progress even after cessation of ITO exposure and has a poor prognosis. To date, no satisfactory effective treatment is available for indium lung disease. These patients usually die from a pneumothorax or respiratory failure. In 2008, indium lung disease is precedent in indium-exposed workers in China. Xiao et al*.* reported pulmonary alveolar proteinosis in a worker exposed to indium as a “sandblaster” used for sputtering of ITO targets while working at a mobile phone manufacturing company [7]. The worker underwent whole lung lavage but died 58 months after diagnosis. Another recent example is the identification of multiple organ injury caused by indium poisoning in a worker who had a history of exposure to metallic indium [8]. With the increasing number of workers engaged in flat panel-display manufacturing, lung diseases related to this occupational exposure are attracting more attention. However, studies focused on indium exposure and the prevention of indium-induced health effects among workers in indium and indium compound handling industries have been limited. The health effects of indium are difficult to assess as a general class because of the diversity of indium and its compounds. Toxicity may vary depending on their different physical and chemical properties. The few human epidemiological studies that have been conducted have typically been small, confined to single ITO workplaces. In view of these knowledge gaps, we selected one representative indium ingot manufacturing plant in southern China and evaluated associations between occupational exposure to indium compounds and human health effects.

The toxicity of indium and distribution in vivo are related to its chemical form, dose, and exposure route. For more realistic modeling of expected serum biomarkers under different exposure and work history scenarios, relevant pathobiology models are needed that account for contributions from all forms of indium encountered in a workplace. In this in-depth study of the mechanism, we hoped to verify the research results from population studies using animal experiments. Therefore, we explored the relationship between the levels of indium exposure and pulmonary injury biomarkers in serum of occupationally indium-exposed workers. Simultaneously, four animal models exposed to different forms of indium compounds [ITO, indium oxide/In_2_O_3_, indium sulfate/In_2_(SO_4_)_3_, and indium trichloride/InCl_3_] were established to detect the changes in the expression of surfactant protein of rat lung tissues, analyze the correlation between them, and elucidate the pathophysiology of various indium compounds exposure. Furthermore, the possibility of surfactant protein as serum markers of indium exposure was explored.

Our study lacked data from ITO manufacturing workers; thus, in the animal experiment part, we established an ITO rat model. The risk of indium lung disease in the industries responsible for manufacturing ITO targets is now well-established. However, more information is needed to understand the risk to workers in other indium compounds industries. Specifically, the results of the rat models established using four forms of indium compounds could help elucidate a number of critical issues. For example, are markers of exposure and health in indium-exposed workers similar to those seen in rat models? Or do these outcomes and therefore risk of indium lung disease vary by the forms of indium? If worker exposure to indium and its compounds in industries is similar to that in rat models of four forms of indium compounds, the study will potentially have far-reaching implications. The purpose of this study is to compare and analyze the causal perspectives of changes in human serum biomarkers induced by indium compound exposure and indium exposure-related rat lung pathobiology, and to discuss possible avenues for their recognition and prevention.

## Methods

### Workers study

#### Study population

The study was designed as a cross-sectional survey. As previously described [9, 10], workers were recruited from an indium ingot production plant (Guangxi, China) that produced indium metal, In_2_(SO_4_)_3_, In_2_O_3,_ and to a lesser extent, InCl_3_. Workers were designated as indium workers based on the potential for exposure to indium in the manufacturing process. A total of 57 indium-exposed workers from eight different types of jobs who were exposed to indium for at least 6 h daily over a period of one year were recruited for the study. On the other hand, the control groups was 63 workers derived from another nearby factory, who were office workers and had no history of occupational exposure to indium and other metals. These participants were matched by sex, age, and work experience to the exposed group. After explaining the purpose and procedure of the study to all participants, their informed consent was obtained. All subjects filled in a questionnaire on demographic characteristics, intake of medication, smoking habits, comorbidity (i.e., the history of respiratory health, hypertension, diabetes, renal and/or urological problems), and working conditions (work tasks and department of work). The exclusion criteria of both groups were: (1) history of respiratory health problems (a medical history of respiratory disease with hospitalization); 3 indium-exposed workers and 4 controls were excluded owing to oral cold or pneumonia drugs; one control with a family history of asthma were excluded. (2) history of taking medications affecting pulmonary and liver function tests; (3) history of exposure to pulmonary toxic chemicals (The indium-exposed workers had no other present or previous exposures to chemicals or particles that are harmful to respiratory health). Spirometry was performed using a Vmax22 spirometer (USA). The parameters measured included forced vital capacity (FVC), lower forced expiratory volume measured in 1 s (FEV_1_), and the FEV_1_/FVC (%FEV_1_ /FVC) ratio.

#### Exposure assessment

The methods used to assess exposure to indium have been described elsewhere [9, 10]. Based on the eight different types of jobs, eight different locations in the workplace were identified as the monitoring sites. Personal breathing zone air samples for each site were collected over three midweek days to measure elemental indium, a marker of indium compounds. The workplace air was drawn at a flow rate of 1.5–3 L/min for a sampling period of 6 h or 8 h, using a Model BFC-35 pump equipped with a mixed cellulose ester membrane filter (a diameter of 37 mm, a cut size of 0.8 μm). On one of the days, workers’ blood and urine samples were also collected. The samples were collected for two more times at each monitoring site. Workers who wore personal sampling devices were asked to record the job tasks during their work periods. After sampling, the collected air samples were preserved at 4 °C until the time of analysis. The limit of detection for airborne indium was estimated to be 0.045 μg/m^3^.

#### Specimen collection and processing

Data collection was completed either at the Institute of Industrial Medicine of the North China University of Science and Technology or at the study plants using previously reported methods [9, 10]. Data and biological specimens collected included answers to a standardized questionnaire on demographics, medical, and occupational history and blood and urine, which were collected for each subject (n = 120) during the annual occupational medicine examination. The samples were collected between the 3rd and 5th of February 2015 at the end of a work shift. Urine samples were collected in 50 mL polyethylene tubes at the end of the morning operating shift (after at least three hours of exposure). Samples were kept on ice packs in a cold box and immediately transported to the laboratory where they were stored at − 20 °C till analysis. Whole blood (16 mL) was collected from workers' arms, of which 8 mL was used to measure biomarkers for the current analysis. The remaining blood was separated into two 4 mL serum separator tubes for serum analysis and two 4 mL EDTA tubes for plasma analysis. After collection, the serum tubes were inverted three times and the blood was allowed to clot for 30 min. All serum and plasma samples were spun to be fractionated at 3000× *g* for 10 min at 4 °C. The samples were split into aliquots and stored in the field at − 20 °C. Samples were shipped overnight and stored at − 80 °C.

### Animal experimental study

In this study, combined with the actual exposure characteristics of indium-exposed workers, rat models were established using four different forms of indium compounds to comprehensively and systematically explore the toxic effects of indium on lung.

#### Experimental animals and environmental conditions

Eight-week-old male Sprague–Dawley rats, weighing about 180–200 g, were obtained from the Medical Laboratory Animal Center of North China University of Science and Technology, China. The rats were housed in an air-conditioned room (temperature of 20 ± 2 °C, relative humidity of 60 ± 10%) with a 12-h light and 12-h dark cycle environment and free access to food and water. The rats were allowed to acclimatize for 1–2 weeks before starting the experimental protocol and were monitored daily for general health. All experimental procedures were conducted in accordance with the Guidelines of the Animal Care and use Committee at the Laboratory Animals Center, North China University of Science and Technology (approval no. 2017011).

ITO, In_2_O_3_, In_2_(SO_4_)_3_, and InCl_3_ (99.9% purity) were purchased from Sigma-Aldrich (USA). The intratracheal instillation model was selected instead of an inhalation method because the former is an easy and reliable method to identify particle toxicity and to compare responses to different particle types. The administered doses in this study is based on LD_50_, i.e. 1.2 mg/kg (1/50 LD_50_), 3 mg/kg (1/20 LD_50_), and 6 mg/kg (1/10 LD_50_).The rats were grouped randomly as follows:

ITO rat model: control group, 1.2 mg/kg bw-ITO group, 3 mg/kg bw-ITO group, and 6 mg/kg bw-ITO group; 10 rats in each group.

In_2_O_3_ rat model: control group, 1.2 mg/kg bw-In_2_O_3_ group, 3 mg/kg bw-In_2_O_3_ group, and 6 mg/kg bw-In_2_O_3_ group; 10 rats in each group.

In_2_(SO_4_)_3_ rat model: control group, 0.523 mg/kg bw-In_2_(SO_4_)_3_ group, 1.046 mg/kg bw-In_2_(SO_4_)_3_ group, and 2.614 mg/kg bw-In_2_(SO_4_)_3_ group; 10 rats in each group.

InCl_3_ rat model: control group, 0.065 mg/kg bw-InCl_3_ group, 0.65 mg/kg bw-InCl_3_ group, and 1.3 mg/kg bw-InCl_3_ group; 10 rats in each group.

Rats were instilled intratracheally following previously described procedures [11]. In brief, the ITO and In_2_O_3_ suspension were sonicated for 30 min to ensure particle dispersion and mixed with 1 mL of saline. In_2_(SO_4_)_3_ and InCl_3_ are soluble indium salt and were thus directly dissolved in normal saline. For the rats in the four forms of indium compound-model groups, suspension was intratracheally instilled twice per week for 8 weeks, and the recovery period was 8 weeks; rats in the saline group were intratracheally instilled with an equal amount of saline twice per week for 8 weeks. All rats were killed at the 16th week. The bronchoalveolar lavage fluid (BALF) was collected via lavage three times using 5 mL of sterile saline and centrifuged at 1000× *g* for 10 min at 4 °C. Lungs were then removed and washed three times with normal saline for complete blood removal. The right lungs were dissected and used for histopathological staining and immunohistochemistry.

### Determination of indium concentrations

Serum indium (S-In) and urine indium (U-In) were measured by inductively coupled plasma mass spectrometry (ICP-MS, Agilent 7500a, USA) after a 1:19 dilution in a 0.1% Triton X-100: 1% HNO_3_ matrix modifier solution. Lung tissue (0.1 g) from each rat was digested in the microwave digestion instrument (MARS6 CEM, Milestone, USA) by adding 2 mL 65% nitric acid (HNO_3_). The digested samples were diluted to 10 mL using ultra-pure water. The indium concentration in tissues was determined using ICP-MS. Concentrations were measured against an addition-calibration curve. Instrumental conditions were set according to methods published previously [12]. Rhodium was used as an internal standard for the indium measurement. The variability of the methods was < 5%, whilst detection limits in both blood and urine were 0.053 µg/L for indium. The quantitative detection limit of indium was 0.045 µg/g for lung tissue. The quantification of elements in serum and urine was accomplished by constructing six-point calibration standards in the range of 0.1–10.0 µg/L that were prepared freshly. Values are presented after being normalized to uric creatinine. The tissue concentration of indium was calculated using the following equation: [indium] treated tissue (µg/g wet tissue) = [indium] tissue suspension/wet weight of tissue.

### Determination of biomarkers in the serum

#### Pulmonary diseases markers

Levels of surfactant protein A (SP-A), surfactant protein D (SP-D), Krebs von den Lungen-6 (KL-6), and granulocyte/macrophage colony-stimulating factor (GM-CSF) were evaluated with ELISA kits (Product ID: JB812-Ra, JB1292-Ra, JB1136-Ra, JB541-Ra; Shanghai, China) according to manufacturers’ protocols.

#### Inflammation markers

Inflammation markers in human serum were analyzed using human inflammatory cytokines ELISA kits, while inflammation markers in rat BALF were analyzed using rat inflammatory cytokines ELISA kits. Inflammation markers analyzed included nuclear factor kappa B (NF-κB), heme oxidase1 (HO-1), interleukin 1β (IL-1β), interleukin 6 (IL-6), interleukin 10 (IL-10), and tumor necrosis factor a (TNF-α) (Product ID: JB548-Ra, JB164-Ra, JB965-Ra, JB952-Ra, JB975-Ra, JB044-Ra, respectively; Shanghai, China).

#### Oxidative stress markers

The hydroxyproline (HYP), total antioxidant capacity (T-AOC), catalase (CAT), alkaline phosphatase (AKP), glutathione peroxidase (GSH), and malondialdehyde (MDA) assay kits were purchased from Nanjing Jiancheng Bioengineering Institute (Nanjing, China), and the levels of these markers were measured according to standard protocols provided by suppliers.

### Biochemical and hematological analyses

Several standard hematological parameters and serum biochemistry parameters were assessed (for specification see Table 2), using a ProCyte Dx Hematology Analyzer, and Catalyst One Chemistry Analyzer, respectively.

### Histopathology and immunohistochemistry

Five rats from each control group and model group were used for histopathological assessment. The lung tissues sections were placed into embedding cassettes and fixed by immersion in 10% neutral-buffered formalin. The formalin-fixed tissues were routinely processed and embedded in paraffin for histopathological examination. Tissue sections (approximately 5-μm thick) were cut and stained with hematoxylin and eosin (H&E) to visualize inflammatory infiltrates. Furthermore, the embedded lung tissues were cut into 3-μm thick sections and stained with Masson’s trichrome to visualize lung fibrosis and periodic acid Schiff (PAS) stain to visualize alveolar proteinosis. Tissue sections were evaluated by a board-certified veterinary pathologist using light microscopy.

Immunohistochemistry for SP-A, SP-D, KL-6, GM-CSF, NF-κB, and HO-1: For immunostaining, lung sectios (5 µm-thick) were deparaffinized and heated in 0.01 mol/L citrate buffer solution (pH = 6.0) for 15 min in a microwave oven for antigen retrieval. The activity of endogenous peroxidases was quenched by applying 0.3% H_2_O_2_ to the sections. A Vectastain rabbit blocking reagent was used to prevent nonspecific binding. Antibody binding was visualized using the avidin–biotin complex (ABC kit, Vector laboratories). Sections were then conjugated, and 3, 3-diaminobenzidine was used as the chromogen to visualize the immunoreaction. Mayer’s hematoxylin was used for counterstaining. All procedures were performed according to the instructions of the Vectastain Elite ABC Kit (Rabbit IgG, catalogue number PK-601, Vector Laboratories, Burlingame, CA, USA). The nucleus, cytoplasm, or cell membrane of positive cells showed brownish yellow staining, and the distribution of positive cells and the expression of target proteins were observed with a light microscope (Olympus Dp25, Japan). The labeling index of SP-A, SP-D, KL-6, GM-CSF, NF-κB, and HO-1 was expressed as the percent positive area/mm^2^ based on Image J analysis (NIH, USA).

### Statistical analysis

The preliminary goals of the analysis were: (1) to compare two indium external exposure measures (airborne indium, job duration), two indium internal dose biomarkers (S-In and U-In), and five pulmonary damage markers (SP-A, SP-D, KL-6, GM-CSF, and pulmonary function) as predictors of pulmonary outcomes in indium-exposed workers and controls, while controlling for covariates; and (2) to compare six oxidative stress markers (HYP, T-AOC, CAT, AKP, GSH, and MDA), and six inflammation markers (NF-κB, HO-1, IL-1β, IL-6, IL-10, and TNF-α) in serum of indium workers and controls, while controlling for covariates.

Descriptive statistics (medians, means, standard deviations, etc.) were calculated for each data type. The normality of all data was tested by the Shapiro–Wilk test. Data normally distributed were expressed as mean ± standard deviation (SD), while skewed data were expressed as median (P_25_-P_75_). Differences in categorical parameters were tested by the Chi square test. Comparisons of medians and means of continuous parameters between exposed and control groups were tested using Wilcoxon rank-sum test and student’s t-test, respectively. All exposed workers were further divided into three subgroups (low, medium, and high duration of exposure) by 25 and 75% percentiles of the working age. Comparisons of medians and means between subgroups were tested by the Kruskal–Wallis H test (when data were not normally distributed) and One-way variance (ANOVA) with least significant difference or Dunnett’s T3 tests, respectively. Multiple linear regression and logistic regression analyses were performed to investigate the differences of biomarkers between groups and subgroups after adjusting for age, sex, body mass index (BMI), and smoking and drinking status. Statistical analysis was performed using SPSS 21.0. Comparisons between groups were performed by applying one-way analysis of variance (ANOVA) followed by Bonferroni’s post-hoc test. *P*-values were obtained from two-tailed tests, and *P* < 0.05 was considered indicative of statistical significance.

## Results

### Workers study

#### Descriptive information for workers and controls

**Fig. 1:**
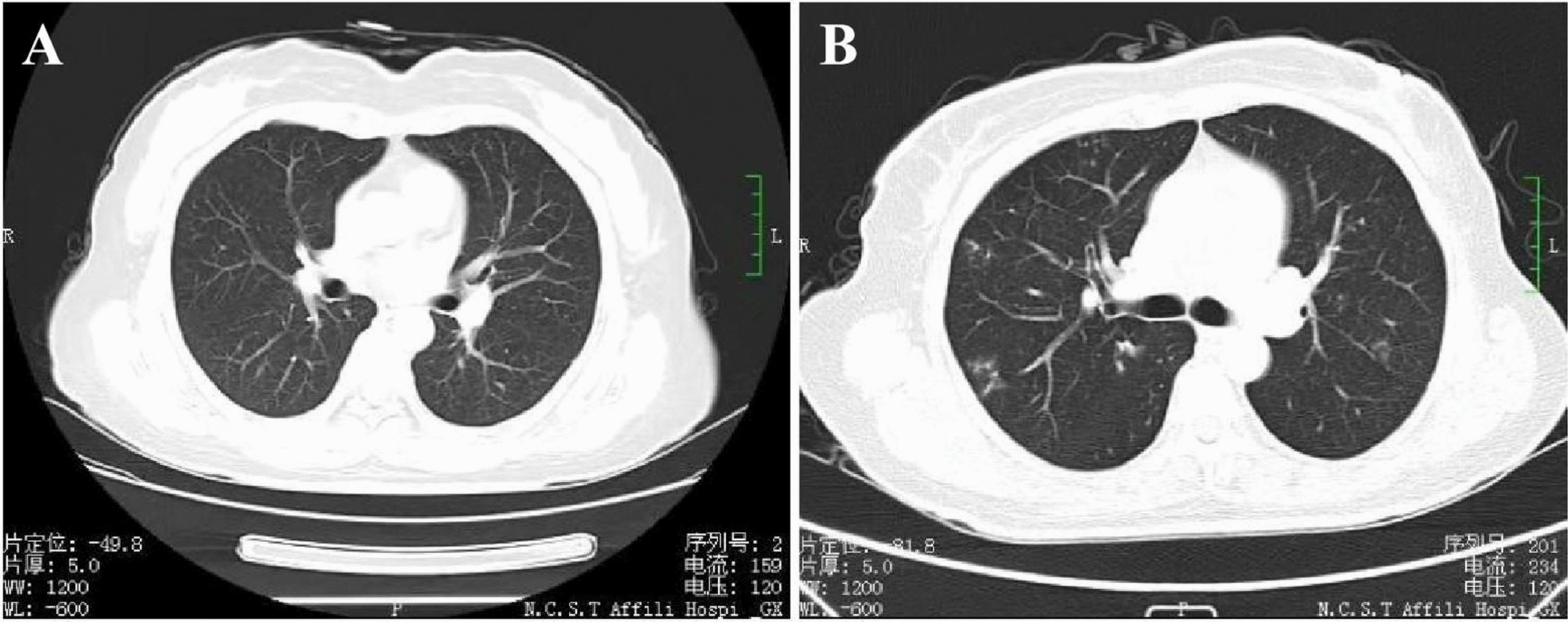
High-resolution computed tomography scans. **A** Example of no pulmonary interstitial changes in controls, show clear subpleural area and no reticular opacities. **B** Example of mild pneumonia in indium-exposed workers, show fine reticulonodular shadows. He had no history of cardiopulmonary disease and no abnormal findings onphysical examination. Results of health examinations were as follows: serum indium, 45.35 μg/L; KL-6, 6383 U/L; FVC, 84.4% predicted; FEV1/FVC, 115.19% predicted

The inclusion criteria of the subjects are provided in our previously published study [9, 10]. A total of 120 study participants comprising of 57 indium-exposed workers (41 males and 16 females) and 63 controls (46 males and 17 females) were recruited for this study. Detailed information on characteristics of all subjects is shown in Table 1. All 120 participants completed the questionnaire, lung function testing, serum indium and urine indium analysis. Among them, 54 workers (45%, 21 controls and 33 indium-exposed workers) agreed to undergo chest high-resolution computed tomography (HRCT). The questionnaire assessed chest symptoms and included questions regarding symptoms of cough, sputum, chest discomfort, palpitation, and wheezing. Forty one of the 57 workers did not experience any of these symptoms, whilst 16 of the workers experienced at least 1 of the 5 chest symptoms. Three workers reported a diagnosis of mild pneumonia; none had radiologic evidence of interstitial pneumonia or pulmonary alveolar proteinosis (Fig. 1). Eleven of the controls experienced at least 1 of the 5 chest symptoms, whilst none had radiologic evidence of interstitial pneumonia or pulmonary alveolar proteinosis. There were no significant age, sex, BMI, and blood pressure differences between the indium-exposed workers and controls, which indicated the basic demographic information of subjects were balanced and comparable. Mean of job duration in years of indium-exposed workers was 8.31 ± 7.54 years (ranged 1–24 years). Although the mean FEV_1_% was within the normal range of > 80%, the observed value of FEV_1_ had significant reductions of 4.4% in indium-exposed group compared with that of the controls (*P* < 0.05). The results from multiple linear regression analysis showed that concentrations of serum indium (*P* < 0.01) and urine indium (*P* < 0.01) were positively associated with airborne indium. The level of exposure to total indium was in the range of 1 to 1120 μg/m^3^, and the values of mean and median of the level of exposure were 78.41 ug/m^3^ and 8 μg/m^3^, respectively. No biological sample gathered from the control group had a quantifiable level of indium. The concentrations of S-In and U-In in indium-exposed workers were (39.26 ± 3.39) μg/L and (11.00 ± 1.67) ng/g creatinine, both statistically significant higher than those in controls [(4.93 ± 0.35) μg/L, (2.23 ± 0.25) ng/g creatinine, respectively] after adjusting for age, sex, BMI, and smoking and drinking status.

**Table 1:** Characteristics of subjects of in controls and indium-exposed workers in China

Characteristic	Controls (n = 63)	Workers (n = 57)	*P*
Age (years)^a^	39.32 (18–60)	37.82 (22–59)	0.44
Sex n (%)^b^			0.89
Male	46 (73.02)	41 (71.93)	
Female	17 (26.98)	16 (28.07)	
BMI (kg/m^2^)^c^	23.62 (15.5–37.8)	24.50 (15.0–39.0)	0.46
FVC (%)^c^	95.42 (80.20–129.60)	91.03 (80.20–125.60)	0.384
FEV_1_ (%)^c^	95.63 (54.20–80.60)	91.46 (80.40–109.60)	0.023^e^
FEV_1_ /FVC (%)^c^	102.49 (81.48–117.32	100.60 (80.55–117.12)	0.588
Systolic blood pressure (mm Hg)^a^	124.59 (101–151)	125.40 (96–166)	0.65
Diastolic blood pressure (mm Hg)^a^	74.90 (55–97)	74.63 (52–106)	0.53
Smoking n (%)^d^			0.17
Yes	31 (49.21)	21 (36.84)	
No	32 (50.79)	36 (63.16)	
Drinking n (%)^d^			0.98
Yes	20 (31.75)	18 (31.58)	
No	43 (68.25)	39 (68.42)	
Job duration (years)	–	8.31 (1–24)	
Airborne indium (μg/m^3^)	–	78.41 (1–1120)	
Serum indium (μg/L)^f^	4.93 ± 0.35	39.26 ± 3.39	< 0.001
Urine indium (ng/g creatinine)^f^	2.23 ± 0.25	11.00 ± 1.67	< 0.001

^a^Data represent Mean (range) and *P* value was calculated by Wilcoxon rank-sum test

^b^One-way ANOVA

^c^Data represent Mean (range) and *P* value was calculated by multiple linear regression analysis

^d^χ^2^ test

^e^Significant difference compared to the control group (*P* < 0.05)

^f^Comparison of medians was performed by multiple linear regression analysis after adjusting for age, sex, BMI, smoking, and drinking status (*P* < 0.001)

#### Serum biomarkers of all subjects

**Fig. 2:**
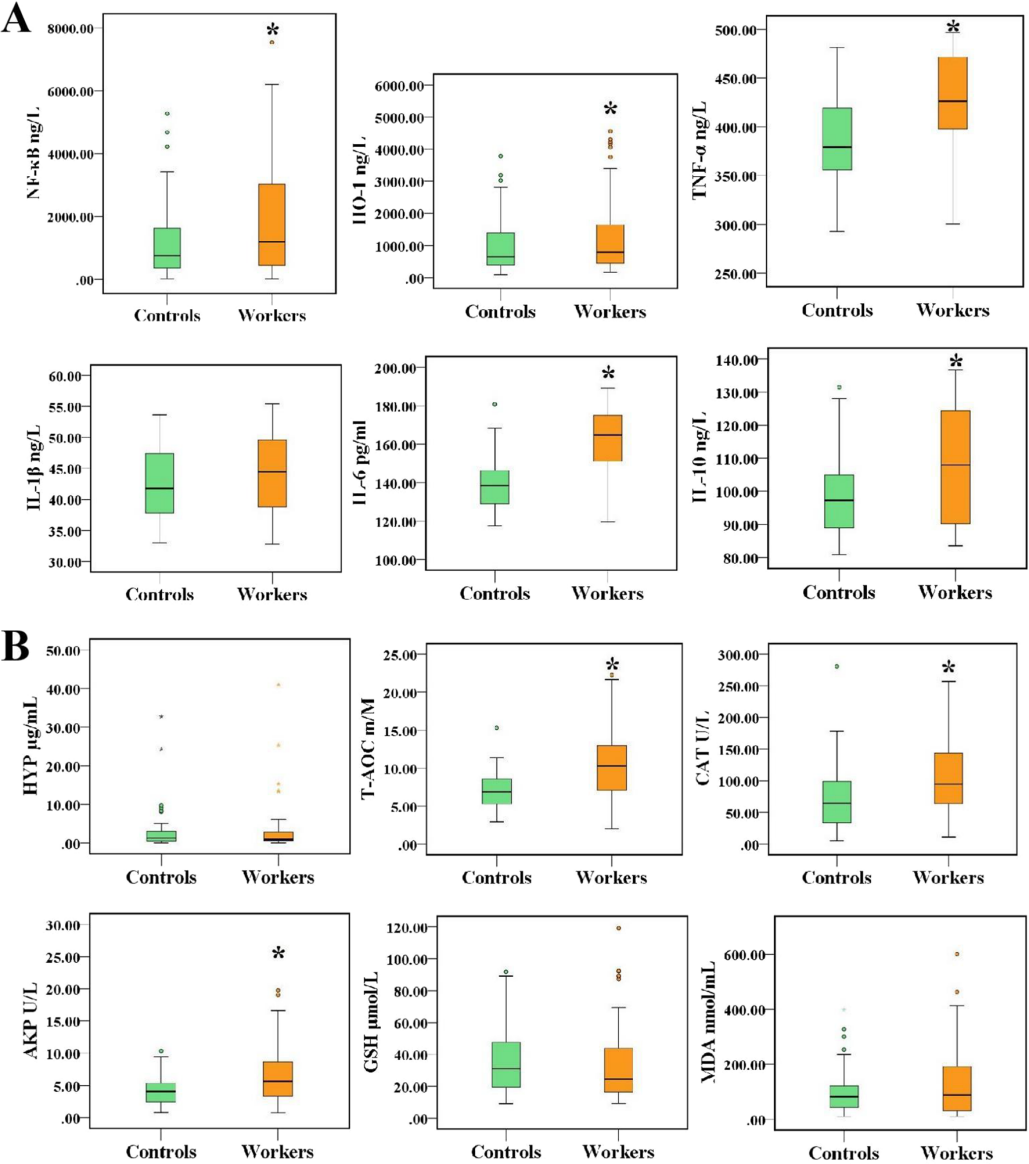
Comparisons of serum biomarkers between workers and controls. **A** Comparisons on inflammation markers. **B** Comparisons on oxidative stress indicators. Comparison of medians was performed by multiple linear regression analysis after adjusting for age, sex, BMI, smoking, and drinking status. * *P* < 0.05

The previous study of our research group found that the levels of SP-A, SP-D, KL-6, and GM-CSF in indium-exposed workers were all statistically significant higher than that in controls (*P* < 0.05) [10]. In this study, we continued to explore the levels of inflammatory cytokines and oxidative stress markers in serum. After adjusting for age, sex, BMI, and smoking and drinking status, concentrations of NF-κB, HO-1, IL-6, IL-10, and TNF-α were found to have significantly increased by 1.6, 1.2, 1.3, 1.1, and 1.1 folds, respectively, in indium-exposure workers, compared with those in control group (*P* < 0.05, Fig. 2A). As shown in Fig. 2B, the level of T-AOC, CAT, and AKP also increased statistically in the indium exposure group [10.30 (7.01–13.05) m/M, 94.62 (62.61–145.96) U/mL, 5.63 (3.31–9.12) U/L] compared with those in the control group [6.88 (5.28–8.64) m/M, 64.31 (28.94–99.40) U/mL, 4.07 (2.34–5.37) U/L, *P* < 0.05] indicating that indium exposure could induce oxidative stress.

#### Hematological and biochemical analysis among all subjects

Some changes were observed in hematological and biochemical markers. Study participants who were exposed to indium had significantly increased medians of RBC, HGB, HCT, MXD%, ALT, AST, and ALB when compared with the non-exposed study participants (Table 2).

**Table 2:** Comparisons on blood hematological and biochemical indicators between two groups

Indicators	Controls (n = 63)	Workers (n = 57)	*P*
*Hematological parameters*			
RBC (10^12^/L)	4.90 (4.57–5.04)	5.01 (4.68–5.24)	0.022^a^
HGB (g/L)	148.00 (137.00–155.00)	156.00 (148.50–162.00)	< 0.001^a^
HCT (%)	0.46 (0.43–0.48)	0.48 (0.45–0.49)	0.012^a^
MCV (fL)	95.10 (92.90–97.20)	95.00 (91.95–97.15)	0.420
MCH (pg)	31.30 (30.50–31.90)	31.40 (30.60–32.10)	0.992
MCHC (g/L)	329.00 (324.00–336.00)	330.00 (326.00–336.50)	0.608
RDW (%)	0.12 (0.119–0.126)	0.12 (0.118–0.125)	0.174
WBC (10^9^/L)	6.80 (6.10–7.80)	6.80 (6.00–7.85)	0.914
LYM%	0.35 (0.30–0.38)	0.34 (0.30–0.37)	0.823
MXD%	0.08 (0.07–0.09)	0.09 (0.08–0.11)	0.003^a^
NEUT%	0.56 (0.53–0.61)	0.56 (0.52–0.60)	0.891
LYM (10^9^/L)	2.30 (2.00–2.60)	2.20 (1.95–2.60)	0.788
MXD (10^9^/L)	0.60 (0.50–0.80)	0.60 (0.50–0.80)	0.566
NEUT (10^9^/L)	3.80- (3.30–4.70)	4.00 (3.30–4.65)	0.811
PLT (10^9^/L)	206.00 (173.00–249.00)	215.00 (185.00–252.00)	0.614
MPV (fL)	9.50 (8.90–10.20)	9.60 (8.95–10.25)	0.931
PDW (%)	11.90 (11.00–13.30)	12.10 (11.00–13.50)	0.684
P-LCR	0.22 (0.19–0.28)	0.22 (0.17–0.28)	0.983
*Biochemical indicators*			
TBIL (μmol/L)	12.80 (10.20–17.50)	13.10 (10.30–16.35)	0.817
DBIL (μmol/L)	1.00 (0.80–1.40)	1.00 (0.70–1.20)	0.457
IBIL (μmol/L)	11.80 (9.60–16.10)	12.00 (9.35–15.35)	0.908
GGT (U/L)	25.0 (18.00–35.00)	32.00 (20.00–43.00)	0.068
ALT (U/L)	21.00 (16.00–33.00)	29.00 (21.50–38.00)	0.001^a^
AST (U/L)	20.00 (17.00–24.00)	22.00 (18.00–30.00)	0.009^a^
TP (g/L)	73.10 (71.60–75.40)	73.90 (72.00–76.50)	0.252
ALB (g/L)	40.36 (36.35–45.37)	52.14 (44.66–62.77)	< 0.001^a^
A/G	2.16 (1.93–2.28)	2.09 (1.88–2.24)	0.264
Serum creatinine (μmol/L)	52.14 (49.46–52.77)	51.71 (46.41–53.57)	0.503

All data were represented as P_50_ (P_25_-P_75_) and *P* value was calculated by multiple linear regression analysis adjusted for age, sex, BMI, smoking, and drinking status

^a^Significant difference compared to the control group (*P* < 0.05)

#### Association between serum indium and health effect biomarkers

Significant positive correlations we observed between serum indium and urine indium, SP-A, KL-6, IL-1β, IL-6, IL-10, TNF-α, TAOC, AKP, RBC, HGB, HCT, GGT, ALT, AST, ALB levels (*P* < 0.05) (Table 3). We further performed multivariate regression analysis (Table 4). In multivariate regression analysis, marginally significant positive relationships were found between serum indium and urine indium levels (*P* < 0.001), SP-A levels (*P* = 0.037), IL-1β levels (*P* = 0.043), IL-6 levels (*P* = 0.008), PDW levels (*P* = 0.029), TP levels (*P* = 0.017). These results implied that exposure to indium may affect the hematological variables, oxidative damage indexes, inflammation factors, and surfactant proteins markers in the indium-exposed workers.

**Table 3:** Correlation and regression coefficients between serum indium and markers of blood biochemistry, oxidative damage, inflammation, and surfactant proteins

Dependent variables	Correlation coeffificients^a^ r (*P* value)	Regression^b^ β (*P* value)	Dependent variables	Correlation coeffificients^a^ r (*P* value)	Regression^b^ β (*P* value)
Urine indium	0.798 (< 0.001)^c^	0.453 (< 0.001)^c^	MCH	− 0.035 (0.352)	− 0.005 (0.974)
FVC	− 0.177 (0.026)^c^	0.044 (0.623)	MCHC	− 0.018 (0.424)	− 0.056 (0.403)
FEV_1_	− 0.141 (0.062)	− 0.012 (0.886)	RDW	− 0.105 (0.126)	0.044 (0.413)
FEV_1_ /FVC	− 0.052 (0.285)	0.021 (0.786)	WBC	0.015 (0.437)	− 0.321 (0.511)
SP-A	0.611 (< 0.001)^c^	0.137 (0.037)^c^	LYM%	− 0.045 (0.311)	0.156 (0.421)
SP-D	0.109 (0.118)	− 0.030 (0.699)	MXD%	0.141 (0.063)	0.024 (0.706)
KL-6	0.220 (0.008)^c^	0.044 (0.486)	NEUT%	− 0.006 (0.475)	0.015 (0.831)
GM-CSF	0.118 (0.101)	0.073 (0.294)	LYM	− 0.015 (0.434)	− 0.042 (0.839)
NF-κB	0.031 (0.366)	0.025 (0.735)	MXD	− 0.022 (0.405)	− 0.017 (0.849)
HO-1	0.111 (0.114)	0.032 (0.683)	NEUT	0.032 (0.363)	0.350 (0.407)
IL-1β	0.189 (0.020)^c^	0.127 (0.043)^c^	PLT	0.040 (0.332)	0.082 (0.253)
IL-6	0.363 (< 0.001)^c^	0.181 (0.008)^c^	MPV	0.056 (0.271)	0.092 (0.864)
IL-10	0.219 (0.008)^c^	− 0.017 (0.791)	PDW	0.105 (0.126)	0.411 (0.029)^c^
TNF-α	0.367 (< 0.001)^c^	0.063 (0.303)	P-LCR	0.049 (0.297)	− 0.466 (0.452)
HYP	0.065 (0.240)	0.026 (0.617)	TBIL	− 0.035 (0.353)	− 0.078 (0.938)
T-AOC	0.302 (< 0.001)^c^	0.021 (0.750)	DBIL	0.017 (0.426)	0.081 (0.220)
CAT	0.147 (0.054)	0.048 (0.385)	IBIL	− 0.038 (0.342)	− 0.075 (0.243)
AKP	0.278 (0.001)^c^	0.094 (0.087)	GGT	0.236 (0.005)^c^	− 0.089 (0.285)
GSH	0.007 (0.469)	− 0.104 (0.155)	ALT	0.346 (< 0.001)^c^	0.166 (0.052)
MDA	0.103 (0.131)	0.019 (0.752)	AST	0.494 (< 0.001)^c^	0.094 (0.244)
RBC	0.318 (< 0.001)^c^	0.011 (0.857)	TP	0.094 (0.154)	0.152 (0.017)^c^
HGB	0.264 (0.002)^c^	0.036 (0.517)	ALB	0.405 (< 0.001)^c^	0.062 (0.396)
HCT	0.372 (< 0.001)^c^	0.063 (0.303)	A/G	− 0.057 (0.268)	0.032 (0.569)
MCV	− 0.002 (0.490)	0.064 (0.628)	Serum creatinine	0.066 (0.238)	0.053 (0.332)

^a^Tested by Pearson test, r (*P* value)

^b^Tested by univariate analysis

^c^*P* < 0.05

**Table 4:** Stepwise regressions between the markers of blood biochemistry, oxidative damage, inflammation, and surfactant proteins

	β coeffificient	*P* value
Model 1: dependent variable: Urine indium	0.500	< 0.001^a^
Model 2: dependent variable: AST	0.250	0.008^a^
Model 3: dependent variable: SP-A	0.258	0.011^a^

^a^*P* < 0.05

#### Exposure time-response relationship between indium exposure and biomarkers

A total of 23 workers exposed to indium for less than 4 years were stratified as low duration of exposure dose group (Group1). Seventeen indium-exposed workers with the working age between 4 and 10 years were classified into medium duration of exposure dose group (Group2) and 17 indium-exposed workers who worked > 10 years were stratified as high duration of exposure dose group (Group3). The general characteristics and serum SP-A of workers in three subgroups are presented in Table 5. The age and job duration showed significant differences between subgroups (*P* < 0.05), while there were no significant differences in other demographic characteristics including sex, BMI, and smoking and drinking status between subgroups. After adjustment for age, sex, BMI, and smoking and drinking status, pulmonary diseases marker SP-A showed a significant rising trend with the increase in working time (*P* < 0.05), and it was the only biomarker with a dose–response relationship among indium-exposed workers in this study (Table 5).

**Table 5:** General characteristics and serum SP-A of subjects in three subgroups

Characteristic	Group1 (n = 23) < 4 year	Group2 (n = 17) 4–10 year	Group3 (n = 17) > 10 year	*P*
Age (years)^a^	29.00 (26.00–39.00)	32.00 (28.00–45.00)	43.00 (40.00–48.00)	0.007^e^
Sex n (%)^b^				0.538
Male	15 (65.22)	12 (70.59)	14 (82.35)	
Female	8 (34.78)	5 (29.41)	3 (17.65)	
BMI (kg/m^2^)^c^	25.04 ± 5.92	24.31 ± 6.10	23.97 ± 5.20	0.835
Smoking n (%)^b^				0.869
Yes	8 (34.78)	6 (35.29)	7 (41.18)	
No	15 (65.22)	11 (64.71)	10 (58.82)	
Drinking n (%)^b^				0.400
Yes	8 (34.78)	7 (41.18)	3 (17.65)	
No	15 (65.22)	10 (58.82)	14 (82.35)	
Job duration (years)^a^	2.00 (1.00–3.00)	7.00 (5.00–8.50)	20.00 (15.00–22.00)	< 0.001^e^
SP-A (μg/L)^d^	103.43 (73.68–164.85)	128.65 (76.70–176.89)	218.45 (94.57–459.95)	0.011^e^

^a^Data represent P_50_ (P_25_–P_75_) and *P* value was calculated by Kruskal–Wallis H test

^b^Calculated by Chi square test

^c^Data were expressed as mean ± SD and *P* value was calculated by One-way variance (ANOVA) with LSD or Dunnett’s T3 tests

^d^Data represent P_50_ (P_25_–P_75_) and multiple linear regression analysis was used to compare values between subgroups after adjusting for age, sex, BMI, smoking, and drinking status

^e^Significant difference between subgroups (*P* < 0.05)

### Study on lung injury induced by exposure to four different forms of indium compounds in rats

#### Characterization of indium compounds

**Fig. 3:**
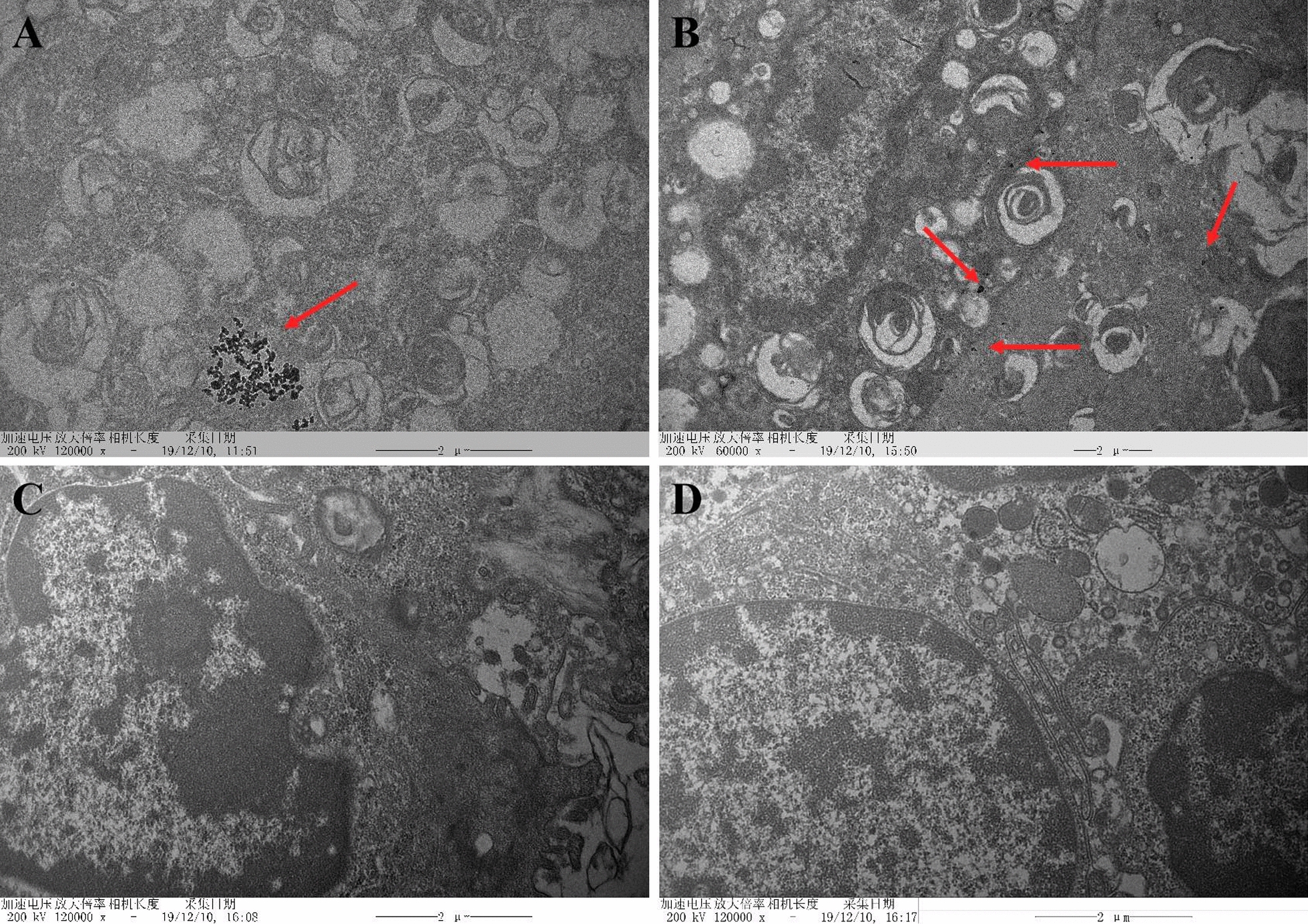
The characterization of four indium compounds. **A** ITO bioaccumulate in lung tissues by TEM analysis (red arrows). **B** In2O3 bioaccumulate in lung tissues by TEM analysis (red arrows). **C** TEM images of lung tissues in rats with In_2_(SO_4_)_3_. **D** TEM images of lung tissues in rats with InCl_3_. Scale bar = 2 μm

Among the four different forms of indium compounds in this study, ITO and In_2_O_3_ are insoluble indium compounds, whereas In_2_(SO_4_)_3_ and InCl_3_ are soluble indium salts. The size and morphological characteristics of ITO and In_2_O_3_ were examined using a high-resolution TEM, and images exhibited a relatively irregular morphology and mild aggregation phenomena (Fig. 3). Meanwhile, TEM revealed ITO aggregates in the lungs of rats exposed to 6 mg/kg bw-ITO (Fig. 3A). Figure 3B shows a TEM image of the presence of dispersed In_2_O_3_ in the alveolar space and alveoli. Figure 3C, D shows TEM images of rat lung tissues after intratracheal instillation of In_2_(SO_4_)_3_ and InCl_3_, respectively. Because of the solubility of In_2_(SO_4_)_3_ and InCl_3_, no particles could be seen in the TEM image of lung tissue. The hydrodynamic size distribution of these four different forms of indium compounds were investigated by particle size analyzer. The hydrodynamic diameter of ITO, In_2_O_3_, In_2_(SO_4_)_3_ and InCl_3_ was (81.09 ± 5.56) μm, (23.14 ± 1.03) μm, (15.36 ± 0.85) μm, and (16.47 ± 0.92) μm, respectively.

#### Body weight

**Fig. 4:**
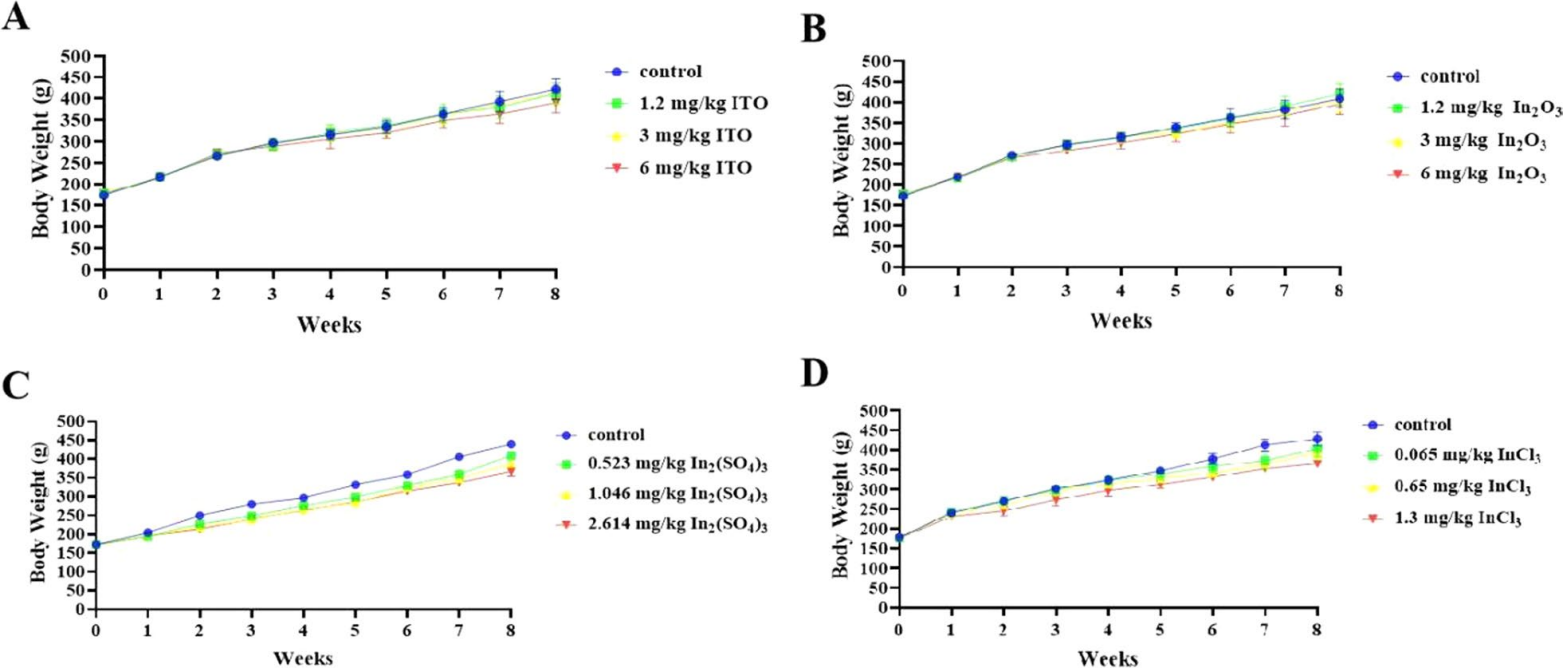
Body weight of the rats in four different forms of indium compounds rat exposure models (n = 10, g). **A** ITO rat model; **B** In_2_O_3_ rat model; **C** In_2_(SO_4_)_3_ rat model; **D** InCl_3_ rat model

No rats died during the intratracheal instillation and observational period. No systemic signs such as respiratory distress, dermatological abnormality, or behavioral or neurological disorder were apparent during the observation period in any group examined. The body weight of rats was continuously recorded for 8 weeks, and the data are shown in Fig. 4. The body weight of four model groups and the control group showed similar increasing trends throughout the study. From the 2nd week, the weight of rats in ITO-exposed group increased more slowly than that in the control group; at the 4th week, the body weight of rats in the 6 mg/kg bw-ITO group was statistically significant lower than that in the control group (*P* < 0.05, Fig. 4A). In the 3rd-5th week of exposure, the body weight of rats in the 6 mg/kg bw-In_2_O_3_ group was statistically significant lower than that in the control group (*P* < 0.05, Fig. 4B). From the first week, the body weight of rats in the 0.523, 1.046, and 2.614 mg/kg bw of In_2_(SO_4_)_3_ group were statistically significant lower than that in the control group (*P* < 0.05, Fig. 4C). After 6–8 weeks of exposure, the body weight of rats in the 0.065 mg/kg bw-InCl_3_ group was statistically significant lower than that in the control group (*P* < 0.05); after the 2nd week, the body weight of rats in the 0.65 mg/kg bw-InCl_3_ group was statistically significant lower than that in the control group (*P* < 0.05); from the first week, the body weight of rats in the 1.3 mg/kg bw-InCl_3_ group was statistically significant lower than that in the control group (*P* < 0.05, Fig. 4D). These results showed that In_2_(SO_4_)_3_ and InCl_3_ had greater effect on the growth of rats compared with ITO and In_2_O_3_.

#### Changes of indium content in serum and lung tissue

**Fig. 5:**
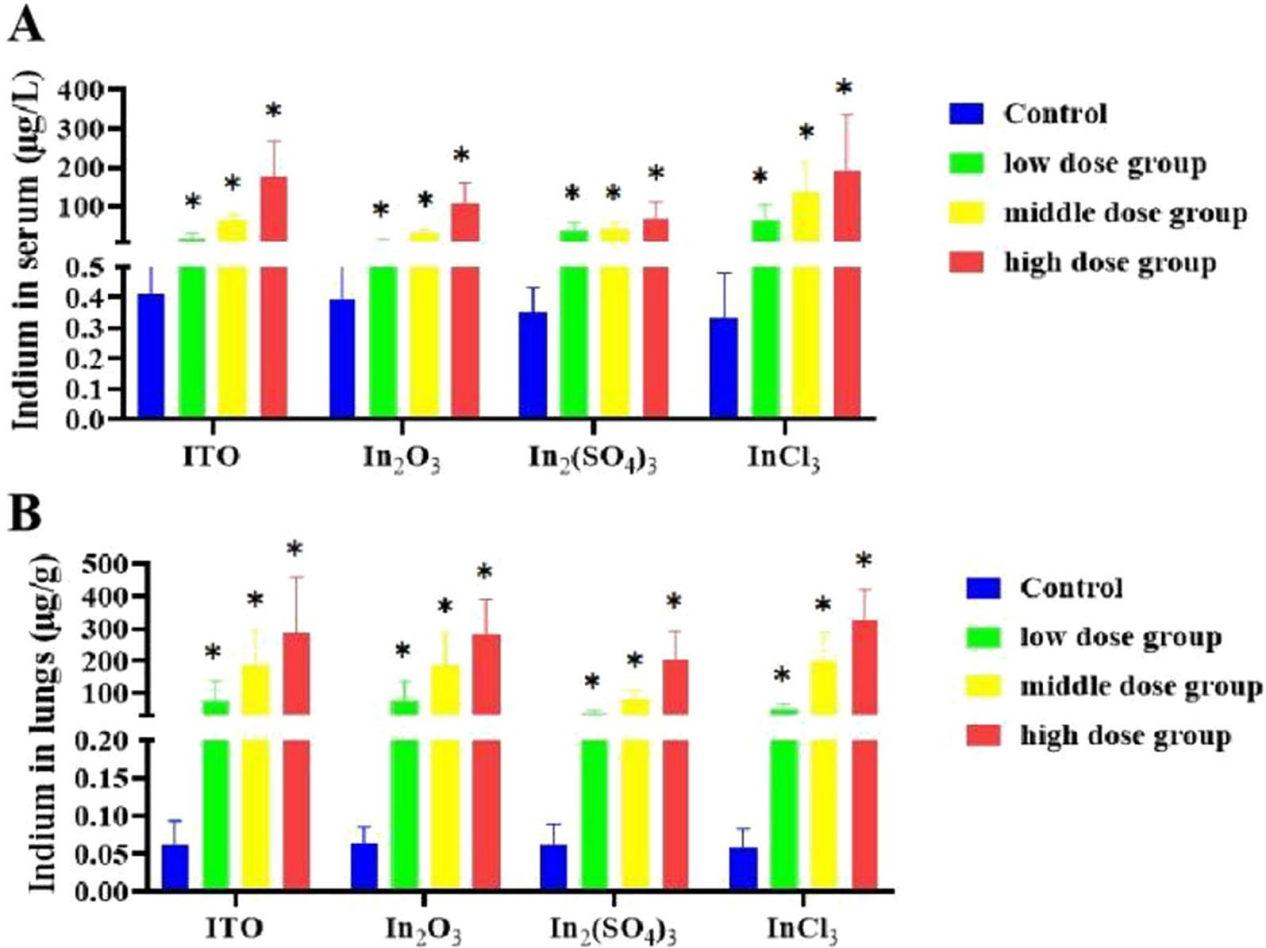
Concentration of serum and lung indium in four different forms of indium compounds rat exposure models (n = 10). **A** Serum indium (μg/L); **B** Lung tissue indium (μg/g)

As can be seen from Fig. 5, contents of serum indium and lung indium in three dose groups of four different forms of indium compounds were statistically significant higher than those in the corresponding control group (*P* < 0.01). Furthermore, the contents of lung indium in all high-dose groups were also statistically significant higher than those in low-dose group (*P* < 0.01). The contents of lung indium in the high-dose group of In_2_(SO_4_)_3_ and InCl_3_ were also statistically significant higher than those in the medium dose group (*P* < 0.05). The contents of serum indium in high-dose ITO and In_2_O_3_ groups were statistically significant higher than those in medium and low-dose groups (*P* < 0.01); the contents of serum indium in all high-dose groups were also statistically significant higher than those in low-dose groups (*P* < 0.05).

#### Analysis of hematology and serum biochemistry

In order to verify the blood index results of indium-exposed workers, blood samples of rat models of four different forms of indium compounds were analyzed for clinical and hematological parameters.

**Fig. 6:**
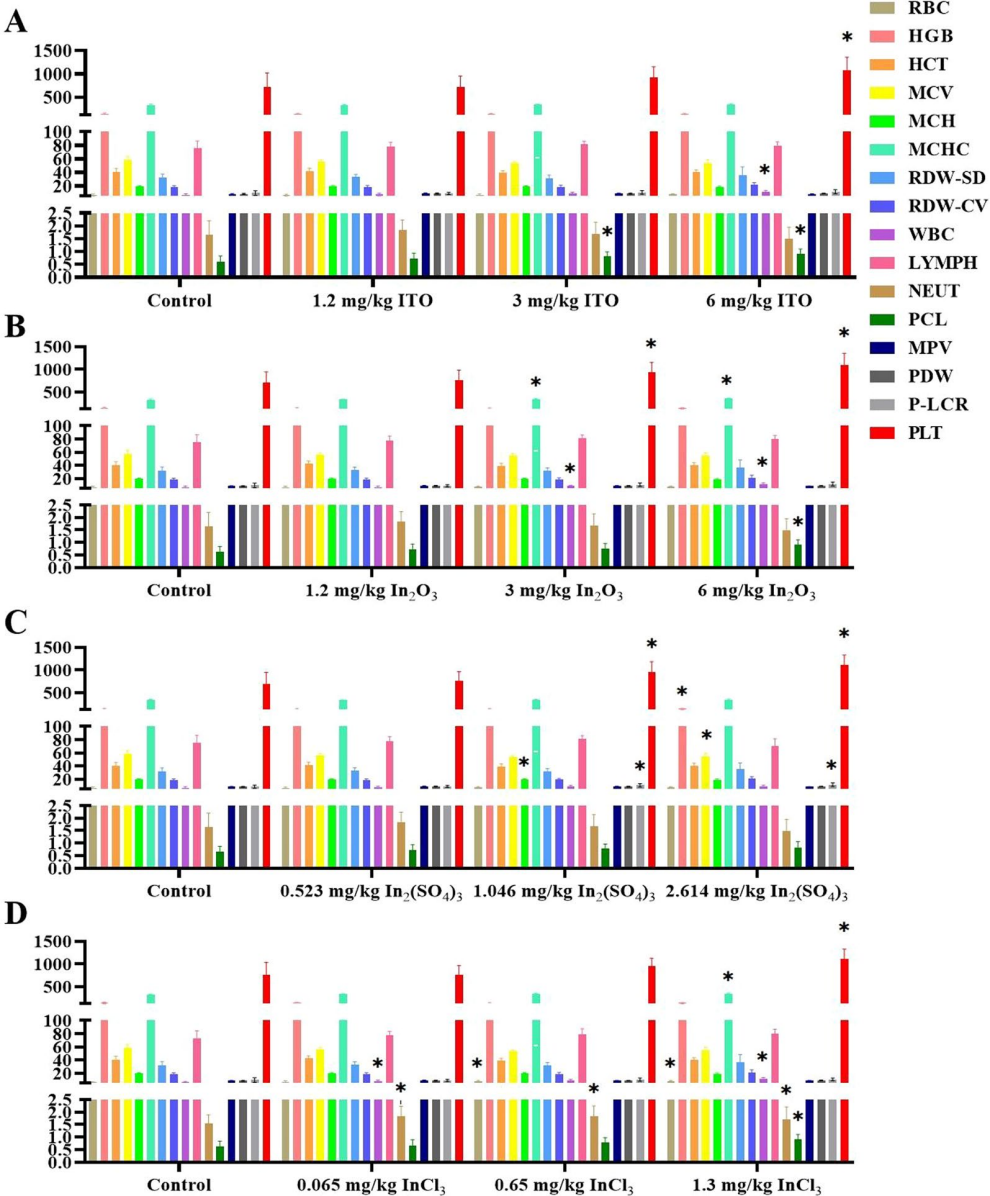
Hematology results in four different forms of indium compounds rat exposure models. **A** ITO rat model; **B** In_2_O_3_ rat model; **C** In_2_(SO_4_)_3_ rat model; **D** InCl_3_ rat mode. Hematology index: RBC levels; HGB levels; HCT levels; MCV levels; MCH levels; MCHC levels; RDW-SD levels; RDW-CV levels; WBC levels; LYMPH levels; NEUT levels; PCL levels; MPV levels; PDW levels; *P*-LCR levels; PLT levels. * *P* < 0.05 vs control

Hematology analysis was performed and the results are shown in Fig. 6. Most hematological parameters were similar between indium compounds-exposed rats and the control group. Compared with the control group, WBC counts and PCL percentage, and PLT count were statistically significant altered in the ITO-exposed rats (*P* < 0.05, Fig. 6A). MCHC levels, WBC count, PCL percentage, and PLT count were statistically significant higher in the In_2_O_3_-exposed rats than those in the control group (*P* < 0.05, Fig. 6B). HGB, MCV, MCH, P-LCR, and PLT counts were higher in high-dose In_2_(SO_4_)_3_-exposed rats than those in the control group (*P* < 0.05, Fig. 6C). Furthermore, RBC, MCHC, WBC, NEUT, PCL, and PLT counts were remarkably higher in the high-dose InCl_3_-exposed rats than those in the control group (*P* < 0.05, Fig. 6D). The above results revealed that high-dose indium compounds exposure can cause changes in hematological indexes.

**Fig. 7:**
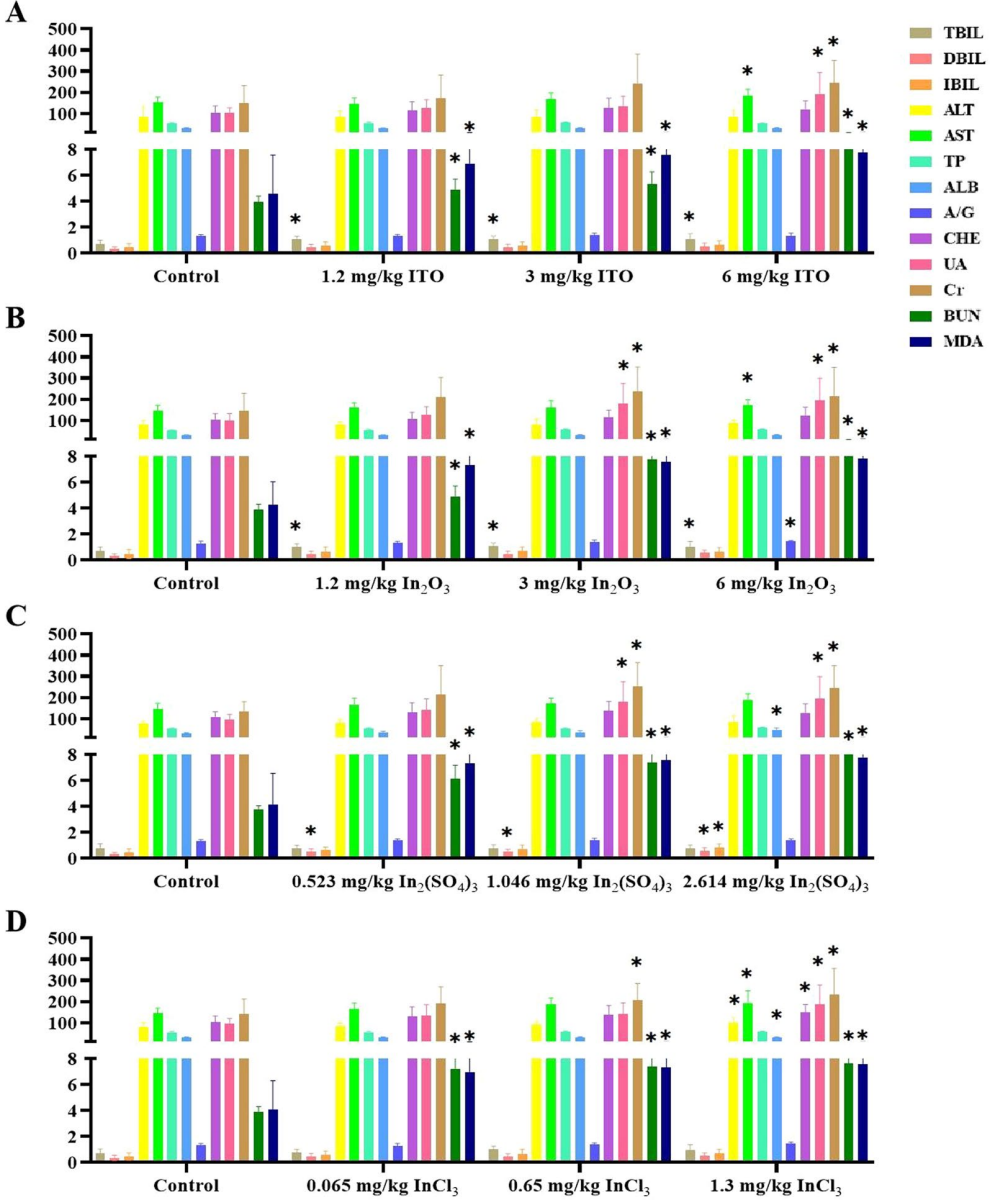
Serum biochemical results in four different forms of indium compounds rat exposure models. **A** ITO rat model; (B) In_2_O_3_ rat model; **C** In_2_(SO_4_)_3_ rat model; **D** InCl_3_ rat mode. Serum biochemical index: TBIL levels; DBIL levels; IBIL levels; ALT levels; AST levels; TP levels; ALB levels; A/G levels; CHE levels; UA levels; Cr levels; BUN levels; MDA levels. * *P* < 0.05 vs control

Serum biochemistry tests were performed to determine the effects of indium compounds on the biological functions of major organs, and parameters including TBIL, DBIL, IBIL, ALT, AST, TP, ALB, A/G, CHE, UA, Cr, BUN, and MDA were measured; the results are presented in Fig. 7. Levels of BUN and MDA were remarkably higher in all indium compounds exposure groups (low, middle, high dose) than in the control group (*P* < 0.05). Moreover, AST, UA, and Cr were statistically significant higher in the high-dose indium compounds exposure groups than in the control group (*P* < 0.05). Furthermore, in high-dose In_2_(SO_4_)_3_ and InCl_3_ groups, levels of ALT, ALB, and CHE were higher than those estimated in the control group (*P* < 0.05, Fig. 7C, D). The results revealed that indium compounds could affect the liver and renal function of rats.

#### Pathological morphology of rat lung tissue

**Fig. 8:**
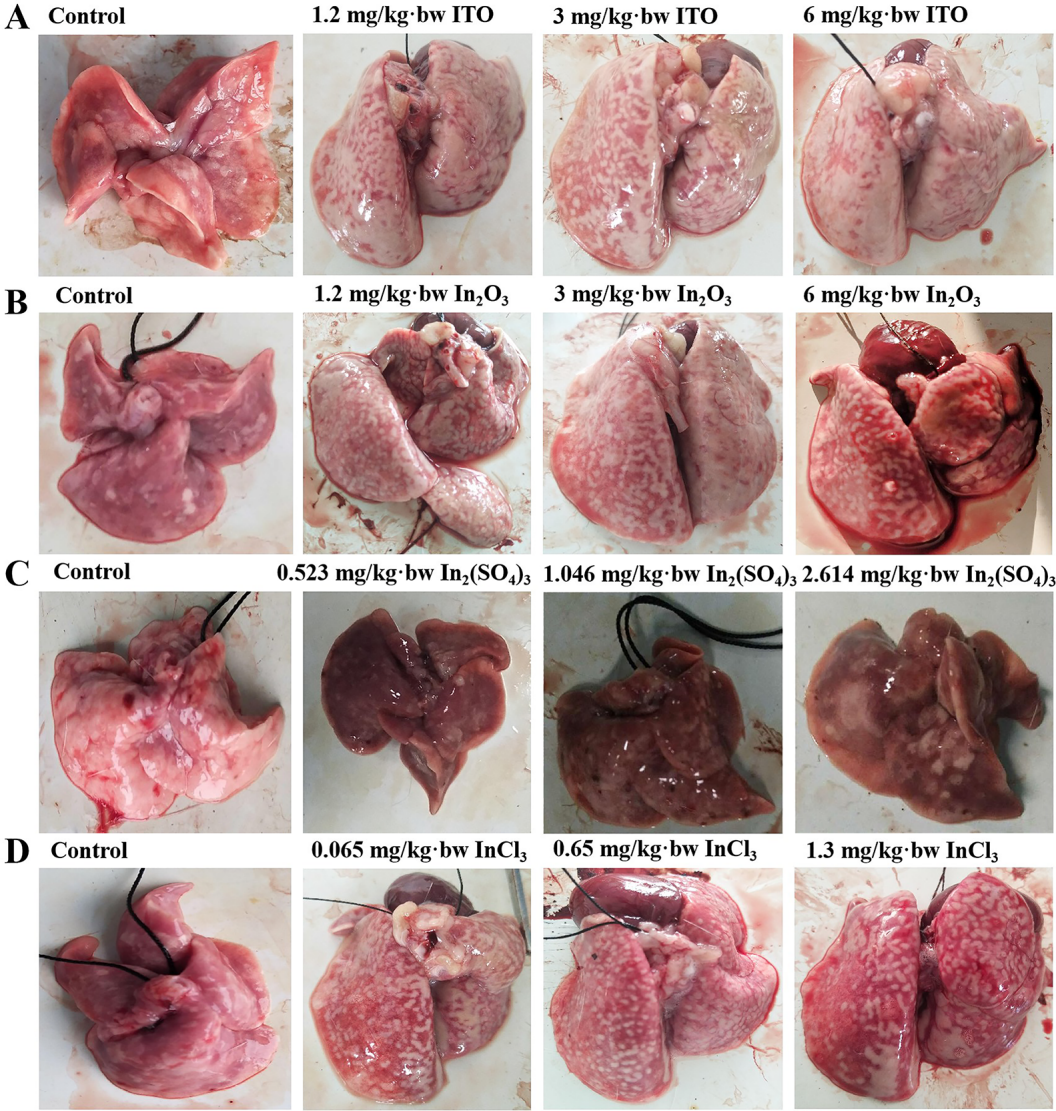
Macroscopic findings in four different forms of indium compounds rat exposure models. **A** ITO rat model; **B** In_2_O_3_ rat model; **C** In_2_(SO_4_)_3_ rat model; (D) InCl_3_ rat mode

In regard to macroscopic findings, the lungs in the control group were soft and elastic, and the surfaces were lustrous. White granular nodules were scattered on the lung surface of the rats in the 1.2 mg/kg bw-ITO and 1.2 mg/kg bw-In_2_O_3_ groups, which worsened with the increase of the exposure dose. The white granular nodules on the lung surface of the rats in the 6 mg/kg bw-ITO and 6 mg/kg bw-In_2_O_3_ groups were almost evenly distributed throughout the lungs; both lungs were significantly swollen and the volume increased; spongy changes and flake bleeding were observed, and punctate white spots appeared (Fig. 8A, B). Compared with other groups, the lungs of rats instilled with In_2_(SO_4_)_3_ showed extensive dark red hemorrhages, indicating that the lungs had severe inflammatory lesions (Fig. 8C). In the 0.065 mg/kg bw-InCl_3_ group, the surfaces of the lungs had white spots and pulmonary hemorrhage. In the 0.65 mg/kg bw-InCl_3_ group, the lungs were congested, swollen, and slightly enlarged. In the 1.3 mg/kg bw-InCl_3_ group, obvious pulmonary edema was observed, accompanied by some dark red hemorrhagic areas and millet gray-white spots on the lung surface (Fig. 8D).

**Fig. 9:**
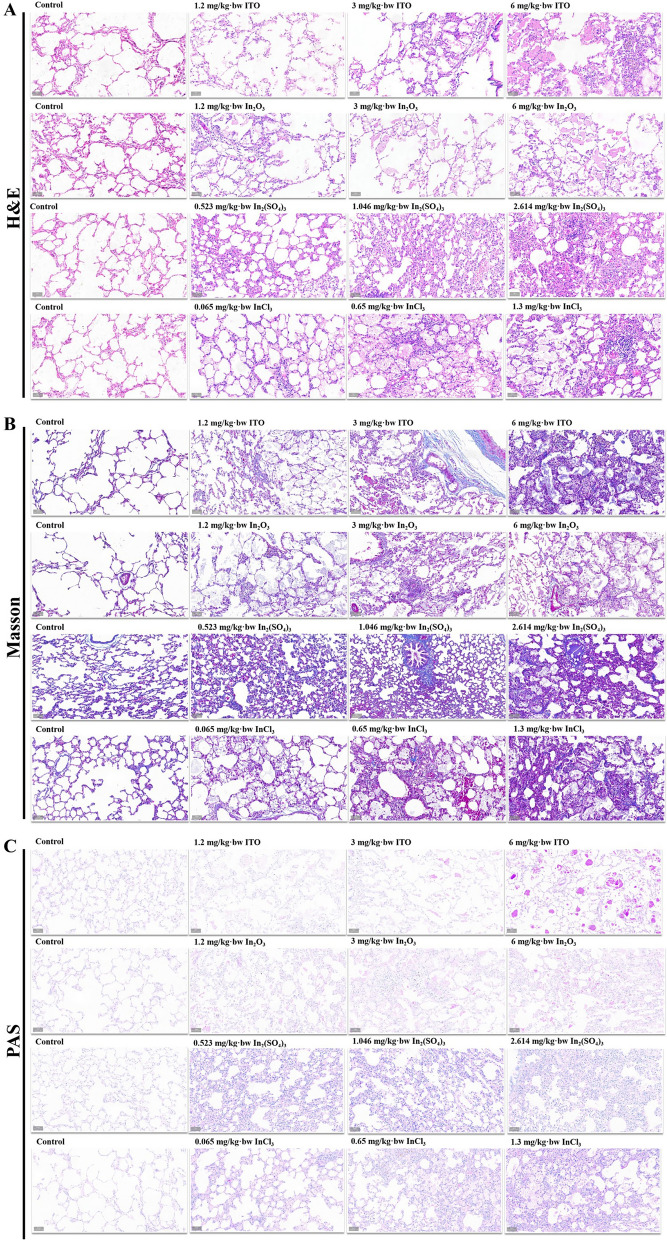
Pathological changes of lung tissue in four different forms of indium compounds rat exposure models. **A** Microscopic findings of the serial lung tissue sections stained with H&E. **B** Microscopic findings of the serial lung tissue sections stained with Masson’s trichrome. **C** Microscopic findings of the serial lung tissue sections stained with PAS. Scale bar = 50 μm

As shown in Fig. 9A, the lung tissue structure in the lung tissue section of the control group was clear, the alveolar structure was complete, the inner wall was smooth, and there was no cell abscission, secretion, and obvious damage. After intratracheal instillation of different doses of ITO and In_2_O_3_, the results of H&E staining showed that the lung tissues of each dosage exposure group were damaged to varying degrees. In the 1.2 mg/kg bw-ITO and 1.2 mg/kg bw-In_2_O_3_ groups, the alveolar structure was relatively intact, the alveolar wall was not thickened, a small amount of uniform eosinophilic fine granular material was deposited in the alveolar cavity, and the alveolar macrophages increased. In the 3 mg/kg bw-ITO and 3 mg/kg bw-In_2_O_3_ groups, the capillaries of the alveolar septum were congested and increase in macrophage proliferation and foam macrophages number were observed. In the 6 mg/kg bw-ITO and 6 mg/kg bw-In_2_O_3_ group, a large number of uniform eosinophilic unstructured fine granular substances could be seen in the alveolar cavity, the alveolar wall was damaged, and the number of foam macrophages increased. The damage in the 6 mg/kg bw-ITO group was more obvious than that in the 6 mg/kg bw-In_2_O_3_ group. In addition to protein like substances, there were also an increase in foam like macrophages that swallow a large number of lipids. After intratracheal instillation of In_2_(SO_4_)_3_, rats in the three dosage exposure groups had different degrees of inflammatory reaction. The alveolar wall structure of rats in 0.065 mg/kg bw-InCl_3_ group was relatively complete, the infiltration of inflammatory cells was less, and the capillaries in the alveolar septum were congested; in 0.65 mg/kg bw-InCl_3_ group, the congestion around the trachea was obvious, and the alveolar septum and bronchiole wall were thickened; in 1.3 mg/kg bw-InCl_3_ group, some alveolar cavities were filled with fine granular protein-like substances, and macrophage proliferation and increase in foam macrophages were observed. In short, a dose-dependent inflammatory response was observed for all four compounds, but that the inflammatory response, for a given dose, tended to be stronger for the soluble than non-soluble compounds.

3 mg/kg bw and 6 mg/kg bw-ITO exposure caused lung fibrosis, which was reflected by the thickening of alveolar septa and subepithelial areas of bronchi and bronchioles. Masson’s trichrome staining revealed a marked increase in fibrous tissue in the lung interstitium (Fig. 9B). Furthermore, lymphocyte infiltration could be observed. Focal chronic inflammation and interstitial fibrosis were observed in some areas of the 6 mg/kg bw-In_2_O_3_ group, but to a considerably lesser extent than that in the ITO groups. In contrast, no obvious, or only a mild fibrotic response, were observed in the 1.2 mg/kg bw-In_2_O_3_ and 3 mg/kg bw-In_2_O_3_ groups, although enlarged, particle-phagocytizing macrophages could be detected in rat lungs. After In_2_(SO_4_)_3_ exposure, minimal to mild septal fibrosis was observed, and the occurrence of septal fibrosis was similar across the three dose groups. Interestingly, the InCl_3_-exposed lungs showed higher collagen accumulation similar to those of ITO groups, as measured by Masson’s trichrome staining, with lung sections exhibiting an increasing amount of blue stain with dosage. The above data collectively indicate that ITO and InCl_3_ can induce lung fibrosis in Sprague–Dawley rat. It is noteworthy that these histopathological changes accumulated with increase in dosage.

PAS staining showed that the alveoli of high-dose ITO and In_2_O_3_ exposure groups were filled with fine, eosinophilic, and PAS-positive pink flocculent deposits, which increased with the increase of exposure dose. Cholesterol granulomas and fibroblastic foci were also observed in some parts (Fig. 9C). The most significant injury was pulmonary proteinosis, which was characterized by granular and eosinophilic substances filling the alveolar cavity. No PAS-positive substance was found in the lung tissue of In_2_(SO_4_)_3_ and InCl_3_ exposure groups, indicating that there was no alveolar proteinosis in In_2_(SO_4_)_3_ and InCl_3_ experimental rats (Fig. 11C). ITO and In_2_O_3_ rat models support findings of both pulmonary alveolar proteinosis and interstitial fibrosis, as seen in human indium lung disease.

#### Expression of interstitial pneumonia markers in rat lung tissue

**Fig. 10:**
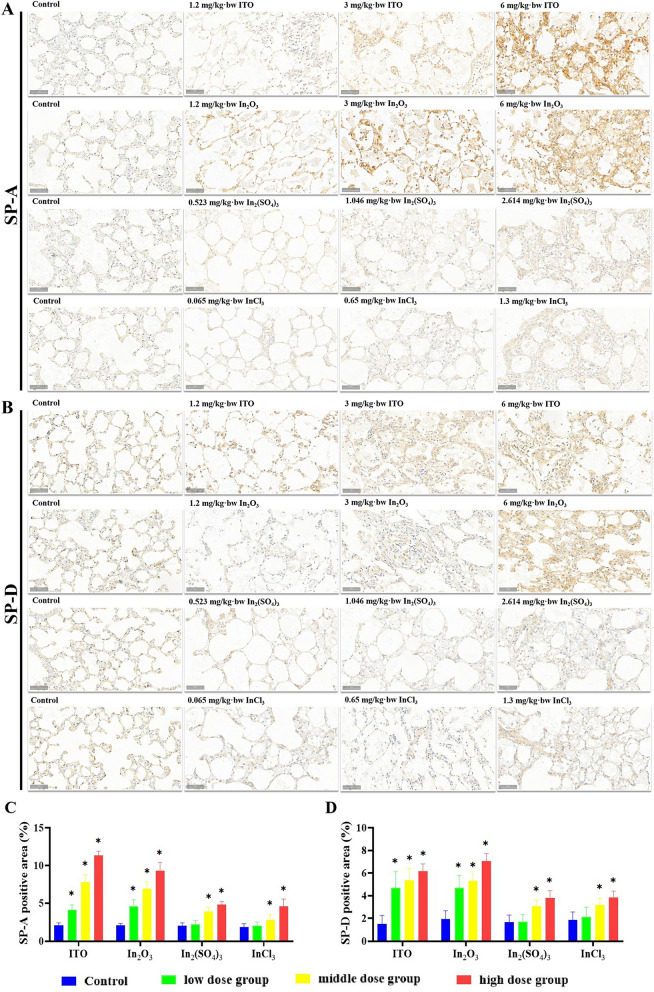

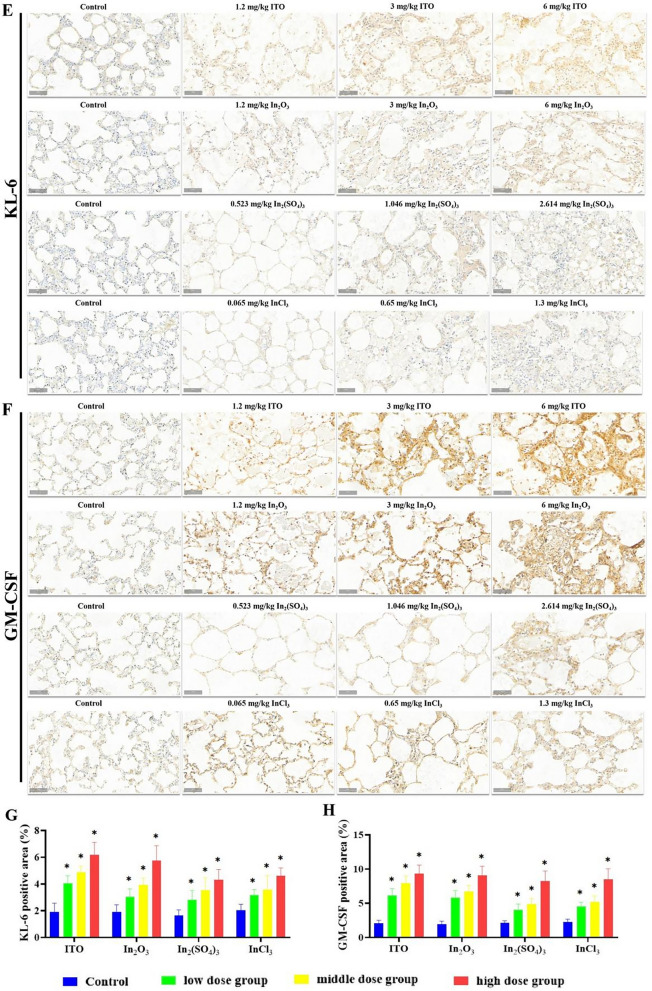
Expression of SP-A, SP-D, KL-6, GM-CSF in the lung of four different forms of indium compounds rat exposure models by immunohistochemistry. **A** SP-A immunoexpression on the lung tissue. **B** SP-D immunoexpression on the lung tissue. **C**, **D** Graph of the mean area % of immunoreaction to SP-A and SP-D. Results are expressed as mean ± S.E. and analyzed using One Way ANOVA followed by Bonferroni’s post-hoc test at *P* < 0.05 (n = 4-6). **E** KL-6 immunoexpression on the lung tissue. **F** GM-CSF immunoexpression on the lung tissue. **G**, **H** Graph of the mean area % of immunoreaction to KL-6 and GM-CSF. Results are expressed as mean ± S.E. and analyzed using One Way ANOVA followed by Bonferroni’s post-hoc test at *P* < 0.05 (n = 4-6). * Indicates significant differences from control rats. Magnification ×200. Scale bar indicates 50 μm

We propose that indium compounds exert cytotoxic effects in the rat lung tissue and damage alveolar type II epithelial cells, and that SP-A, SP-D, KL-6, and GM-CSF are specific biological indicators of lung injury. Immunostaining for SP-A showed that, in the lung exposed to ITO and In_2_O_3_, positive staining of SP-A was observed not only in type II alveolar epithelial cells but also in alveolar macrophages and alveolar mucus (Fig. 10A). The brown-yellow particles in the rat groups exposed to In_2_(SO_4_)_3_ and InCl_3_ were decreased compared to those in the ITO and In_2_O_3_ groups, but the positive staining of SP-A in lung tissue of rats in 1.046 mg/kg bw-In_2_(SO_4_)_3_, 2.614 mg/kg bw-In_2_(SO_4_)_3_, 0.65 mg/kg bw-InCl_3_, and 1.3 mg/kg bw-InCl_3_ groups were significantly increased compared to those in the control groups. SP-D immunohistochemical staining results also showed that more brown-yellow particles were found in rat alveolar type II epithelial cells and in some macrophages in ITO and In_2_O_3_ groups (Fig. 10B), and the results were consistent with those for SP-A. SP-A and SP-D staining performed in ITO and In_2_O_3_ groups showed that granular materials within alveolar spaces were strongly positive for SP-A and SP-D, and indicated the presence of surfactant proteins in eosinophilic materials. Type II pneumocytes surrounding a pulmonary alveolar proteinosis-like change were also positive for SP-A and SP-D. Figure 10E–H shows the effect of four indium compounds exposure for 8 weeks on the expression of KL-6 and GM-CSF in lung tissues. The immunostaining of lung sections demonstrated that the expression of KL-6 and GM-CSF in all compounds groups was statistically significant higher than that in the control group, and the most significant immunoreaction of GM-CSF was observed in ITO and In_2_O_3_ groups.

#### Expression of inflammatory markers in rat lung tissue

**Fig. 11:**
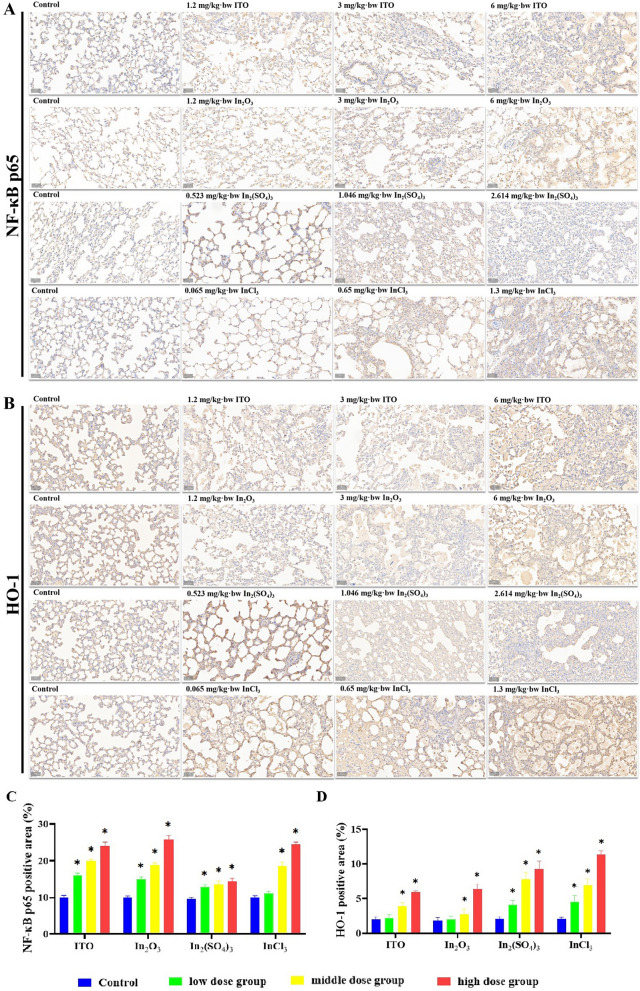
Expression of NF-κB p65 and HO-1 in the lung of four different forms of indium compounds rat exposure models by immunohistochemistry. **A** NF-κB p65 immunoexpression on the lung tissue. **B** HO-1 immunoexpression on the lung tissue. **C**, **D** Graph of the mean area % of immunoreaction to NF-κB p65 and HO-1. Results are expressed as mean ± S.E. and analyzed using One Way ANOVA followed by Bonferroni’s post-hoc test at *P* < 0.05 (n = 4–6). * Indicates significant differences from control rats. Magnification ×200. Scale bar indicates 50 μm

**Fig. 12:**
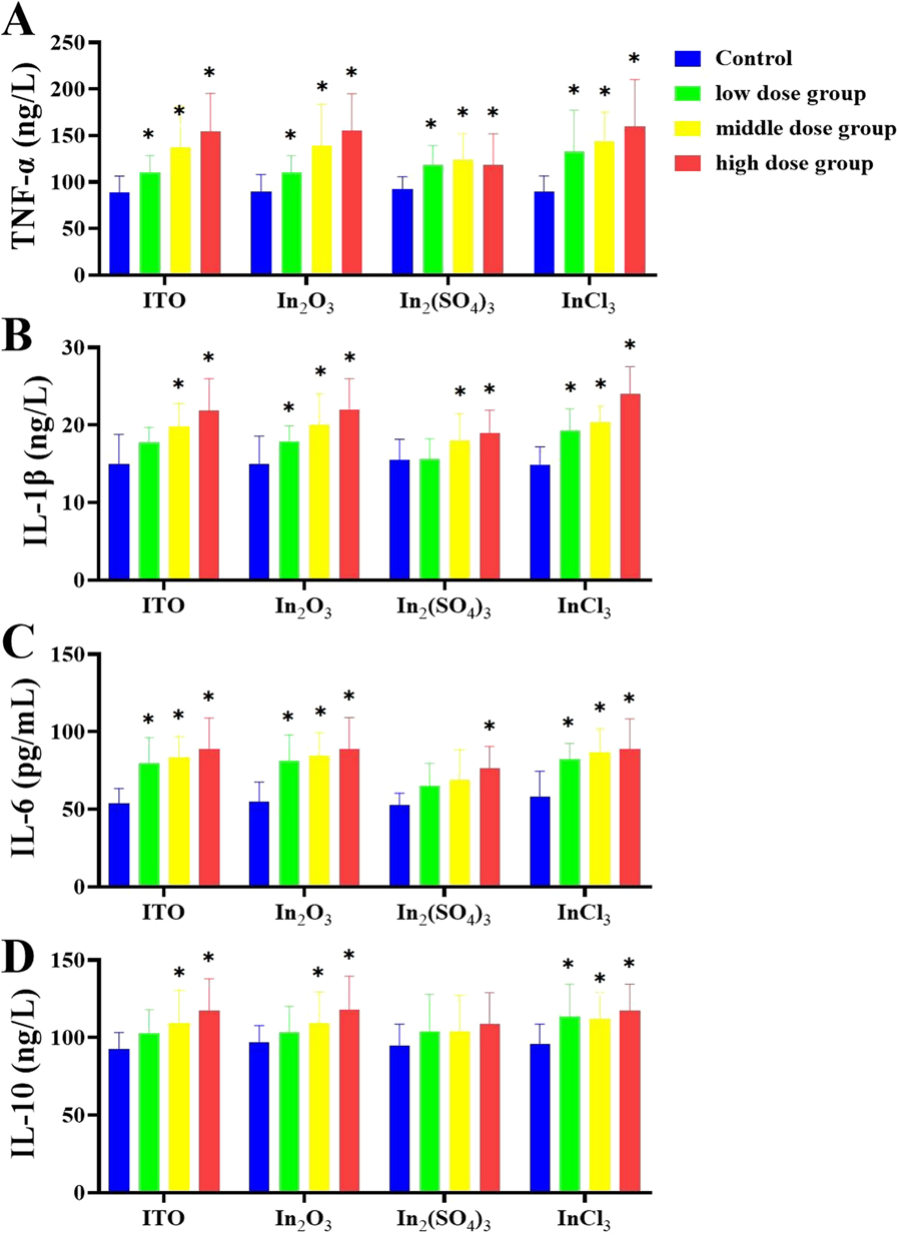
Analysis results of BALF. **A** TNF-α levels; **B** IL-1β levels; **C** IL-6 levels; **D** IL-10 levels. *P < 0.05 vs control

To evaluate whether four different forms of indium compounds could evoke pulmonary inflammation and to compare it with the serum analysis in indium-exposed workers, the expression of NF-κB p65 and HO-1 in rat lung tissues was observed and the levels of IL‑1β, IL‑6, IL‑10, and TNF‑α in the BALF supernatant were detected. Figure 11 shows the effect of four indium compounds exposure for 8 weeks on the expression of NF-κB p65 and HO-1 in lung tissues. The immunostained lung sections demonstrated that the expression of NF-κB p65 and HO-1 in the 6 mg/kg bw-ITO, 6 mg/kg bw-In_2_O_3_, 2.614 mg/kg bw-In_2_(SO_4_)_3_, and 1.3 mg/kg bw-InCl_3_ groups were statistically significant higher than that in the control group; the most significant immunoreaction of NF-κB p65 was observed in ITO and In_2_O_3_ groups, while the most significant immunoreaction of HO-1 was observed in In_2_(SO_4_)_3_ and InCl_3_ groups. ELISA analysis of BALF found that the levels of the pro-inflammatory cytokines were significantly elevated as compared with those in control group (Fig. 12). Although partly less apparent in the In_2_(SO_4_)_3_-exposed group, levels were increased, except for IL-10, showing a clear dose-dependent relationship. A statistically significant difference was observed in results for all groups, except for IL-6 and IL-10 in the In_2_(SO_4_)_3_-exposed group, when compared with those in the control group.

## Discussion

### Study from cross-sectional survey

During its manufacture and use, indium can enter human body through several exposure routes, such as inhalation and dermal contact [6, 7, 13]. Among them, inhalation is the most important route, especially in an occupational setting. Since lungs are likely to be the primary target organ affected by indium, the number of studies focusing on the respiratory system is far larger than that focusing on other exposure routes. It has also been found that dermal exposure to ITO leads to an immune stimulation, with a dose-responsive increase in lymphocyte proliferation observed, which can induce hypersensitivity responses [13]. Numerous metals are known to induce hypersensitivity responses, resulting in varying levels of morbidity, from chronic allergic contact dermatitis to potentially fatal pulmonary disease. Available case reports and epidemiological studies indicate that individuals who inhale indium-containing dusts may develop indium lung disease; however, the disease has not been causally linked to a specific chemical form of indium. Specifically, it is unclear if all forms of indium are capable of inducing pulmonary alveolar proteinosis and fibrosis, and thereby, pose the same disease risk. The subject of this study, was a small indium ingot production plant, where ventilation facilities such as local exhaust systems were not installed in most cases. Workers are exposed to various types of indium or indium compounds in the form of particulates, aerosols, and fumes. In_2_O_3_, InCl_3_, In_2_(SO_4_)_3_, and indium fumes are typical. In this study, FVC, FEV_1_, and FEV_1_/FVC were used as indicators of abnormal lung function in indium-exposed workers, and their associations with serum indium levels were analyzed. Hematology analysis and serum biochemistry analysis are commonly used to evaluate lipid accumulation. Pulmonary damage markers combine three kinds of serum indicators, namely, interstitial pneumonia markers, inflammation markers, and oxidative stress markers, to better reflect the extent of pulmonary injury. The data here strongly suggest that indium could contribute, at least in part, to the pulmonary effects observed among indium-exposed workers.

A large number of epidemiological studies indicated that workers with significantly longer exposure periods had higher serum indium concentration, and the correlation between serum indium concentration and cumulative exposure was better than that of current exposure, which resulted in higher risk of lung disease [14–17]. However, indium concentration alone cannot distinguish between the form of indium (i.e., indium metal, indium salt, indium oxide, or ITO) to which a worker has been exposed, nor can it capture properties such as surface chemistry and solubility that may be relevant to differential toxicity. A better understanding of exposure through the use of more sensitive serum indium tests and promising blood biomarkers of lung inflammation that differentiate between the various indium compounds is also needed, and will play a crucial role in the control of this emerging occupational health hazard. Abnormal pulmonary surfactant protein levels are the main cause of pulmonary alveolar proteinosis. Presently, there are few studies on the effects of indium exposure on serum pulmonary surfactant protein levels in indium-exposed workers, and there is no consensus on the findings. For example, in an 11‐year multicenter cohort study, Nakano et al*.* found that nonspecific serum inflammatory biomarkers of lung damage, including KL-6 and SP-D, were all elevated in the serum of four lung cancer cases exposed to indium compared to non-exposed workers [18]. In a low serum indium level study of ITO workers, Yang et al*.* found serum GM-CSF, IL-4, IL-5, TNF-α, and TNF-β levels were significantly increased, and showed a significant correlation with serum indium levels [19]. Our previous studies demonstrated significant increase of interstitial lung disease markers including SP-A, SP-D, KL-6, and GM-CSF in indium-exposed workers, which gave some insight into the formation of some of the factors derived in our study [10]. In the present study, NF-κB, HO-1, IL-6, IL-10, and TNF-α were the most obvious elevated markers in indium-exposed group compared with the levels in the controls, showing that they may be more sensitive to inflammatory responses induced by indium exposure than other cytokines. There were no significant differences in concentration values of IL-1β between two groups. Nagano *et al.* found that serious lung lesions caused by indium exposures started with pulmonary alveolar proteinosis following inflammation [20–22]. In this study, although the indium-processing workers did not show pulmonary dysfunction, an inflammatory response was induced, which may either be an adaptive response or may further cause chronic lung diseases. Oxidative stress is considered to be an important mechanism responsible for adverse health effects induced by indium. In this population study, the level of T-AOC, CAT, and AKP, were found to increase on indium exposure. The activity of serum T-AOC, an important antioxidant enzyme in protecting body from oxidative damage, increased in the exposed group, indicating oxidative stress in indium-exposed workers. Furthermore, the early biological indicators of interstitial pneumonia (SP-A), inflammation (IL-1β, IL-6), and oxidative stress (AST) were significantly associated with serum indium levels. Taken together, these studies indicated that abnormalities of these inflammatory factors could induce lung inflammation.

Although exposure time is not fully equivalent to exposure dosage, it can reflect workers’ cumulative exposure level in part when considering that the exposure concentrations were relatively similar in different workshops. It is also very important to know whether indium is bioaccumulative, or if it is excreted before the start of the next working day. In this study, we found significant differences in several biomarkers between exposed workers and the controls. Among them, only SP-A showed a time-response pattern among indium-exposed workers. With the increase of job duration, SP-A demonstrated a significant rising trend. This was likely due to the cumulative exposure dosage and lung burden of workers increasing as their exposure time extended, resulting in a further reduction of type II alveolar epithelial cells and/or more severe damage of cell function and structure, eventually leading to a increase in SP-A secretion. This study did not find time-response relationships between other biomarkers and exposure. The possible reasons are that these biomarkers may be associated with exposure in a significant manner, but their changes are acute or non-progressive. In addition, markers other than SP-A may not be sensitive enough to show differences between exposure and dosage. Therefore, we suggest that SP-A should be listed as a key and sensitive health surveillance marker for indium exposure, but more studies are needed to support this suggestion.

In a cohort study, Toshiharu et al*.* found that although there were significant differences in subjective respiratory symptoms, the increase in biomarkers was minimal, with only nine (2.1%) workers showing S-In and KL-6 levels higher than the standard values [23]. This is in contrast to results from previous studies that reported an increased risk of lung cancer and interstitial pneumonia with indium exposure. In an 11-year cohort study [18], the S-In and KL-6 levels were higher in indium-handling workers than in those who were not exposed. Other studies have also reported higher S-In, KL-6, and SP-D levels among indium-handling workers [24, 25]. A cross-sectional study at one site in Korea found higher S-In and KL-6 levels were associated with HRCT-detected interstitial lung changes and demonstrated a significant dose–response relationship between serum indium levels and KL-6 as well as interstitial changes on HRCT [26]. However, in our study, no significant association was found between HRCT changes and indium exposure. This might be influenced by the limited number of the study subjects. Because relatively small number of the workers had volunteered for HRCT, the participants might also be influenced by selection bias. Another possibility is the limitations of the cross-sectional approach, e.g. healthy worker effect when studying currently indium-exposed workers, which could affect possibilities to detect other indications of clinically manifest lung injury like lung function and respiratory symptoms. Nakano et al*.* observed a dose–effect relationship between In-S and KL-6 levels in workers exposed to indium metal [18]. Our previous study found that there was a significant correlation between serum indium concentration and serum SP-A [10]; in this study, we further stratified the duration of exposure and found that with the increase in working time, SP-A showed a significant upward trend (*P* < 0.05). The possible reasons for such differences are that in our study, subjects were exposed to a real-time level of indium during manufacturing process, which was much higher than that of the controlled studies. The mean level of indium in the serum of these workers was 39.26 μg/L, and the lowest level was 11.86 μg/L. All workers had serum indium levels higher than 3 μg/L, the recommended biological exposure index for indium exposure by the Japan Society for Occupational Health. The study by Harvey et al*.* included 80 workers at a single ITO-producing site in America [27]. And the workers included in the study by Cummings et al*.* were only exposed to ITO (0.4 to 108 μg/m^3^) [15]. In contrast, our analysis included more potential confounders as we studied exposure to four different forms, namely indium metal, In_2_(SO_4_)_3_, In_2_O_3_, and InCl_3_. Besides, concentrations of serum indium (*P* < 0.01) and urine indium (*P* < 0.01) were positively associated with airborne indium in our study. Workers in our study were likely exposed to a wider variety of indium types and compounds, including metals, than workers in other studies. This may have concealed associations between exposure to specific types of indium (e.g., ITO) and biomarkers of early effect. We were unable to take into consideration indium type because, as shown in the study by Higashikubo et al*.*, many workers who were exposed to ITO/In_2_O_3_ were also exposed to indium. Similarly, workers who were exposed to ITO were also exposed to indium nitrate [28]. Furthermore, our S-In and U-In analysis was unable to distinguish between ITO, In_2_O_3_, In_2_(SO_4_)_3_, or InCl_3_.

In the population study part, we found consistent associations between serum indium concentration and clinical, functional, and serum biomarkers, suggesting a possible mechanism of inhaled indium inducing inflammatory response via these inflammatory factors and thus causing lung diseases. These cytokines may be predictors of indium lung disease. However, inflammatory responses induced by other causes can also lead to the abnormal expressions of these cytokines. To some extent, these cytokines may act as predictors of indium lung disease with low specificity. There are certain limitations of population studies such as unacceptability of pathological study of lung tissue in the general population, which could be simulated and discussed in animal experiments. Therefore, further follow-up animal experiments need to be carried out for confirmation of these cytokines as predictors of indium lung disease.

### Study from rat models exposed to four different forms of indium compounds

Understanding the relationship between exposure to indium of different forms and pulmonary damage has critical implications for prevention of indium lung disease in current and future indium industry workers. Therefore, to observe the effect and mechanism of action of indium and its compounds on the lungs, as well as to verify the effect of serum biomarker levels observed in the population, we established four rat models through intratracheal instillation exposure to different indium compounds to simulate indium exposure in the occupationally exposed workers, though the method of exposure was not the same. The different kinds of indium compounds in rat models corresponded to the different specific exposure of workers. Prior to this report, little was known about the animal model experiment of real-world indium-containing dusts to which workers are exposed. Our animal experiments covered four different forms of indium (ITO, In_2_O_3_, In_2_(SO_4_)_3_, and InCl_3_), of which three forms of indium compounds were present in various dusts encountered during production at one indium ingot plant. In addition, the four different forms of indium compounds were administered as low, medium, and high doses in the form of NOAEL/LOAEL for the animal study, in order to guide the risk assessor as to what the safe levels are in humans.

To the best of our knowledge, our verification of human serum biomarkers results in rat models of four forms of indium compounds is unique. Notably, protein expression in rat lung tissue was quite comparable to serum biomarkers in workers, demonstrating the same health outcomes and similar relational patterns. Serum biomarkers likely responded to the actual exposures compared to the measured protein concentrations in the rat models. Thus, had we been able to accurately account for the serum biomarkers of indium exposure, we expect that we would have found health effects at an even earlier stage in workers exposed to indium and its compounds.

Despite the four rat models being relatively independent from real-world exposure, the associations we found are likely to be due to the effect of indium exposure on the lungs for several reasons. Firstly, they are biologically plausible, given the lung toxicity of In_2_O_3_ and ITO observed in animal studies. Secondly, they are consistent with the lung disease reported in ITO industry workers, notably, pulmonary alveolar proteinosis [29]. Thirdly, they reflect subclinical or undiagnosed lung disease experienced by indium industry workers in many other facilities worldwide. Fourthly, they were not accounted for by potential confounders such as age or smoking, which are known risk factors for decreased lung function.

Indium and biomarkers reflecting lung injury were correlated, and results were similar between rat models of four forms of indium compounds and serum analysis of indium-exposed workers. These findings are not unexpected, given that most processes in the plant involve working with some form of indium. The agreement between protein expression in rat lung tissue and serum biomarkers in workers suggests that biomarkers of inflammation, oxidative stress, and lung injury in serum may be reasonable surrogate for indium exposure in this plant. This observation has practical consequences, as the detection of biomarkers in serum can be used to identify high exposure tasks that requirie early screening. Serum measurement also can be used as a surveillance tool to quickly, simply, and cost-effectively identify indium-exposed workers. Yet a likely better option is to evaluate work procedures at the plants and make an assessment of exposure risk, and not to wait until exposure has occurred.

Potential indium exposures at this plant were not limited to In_2_O_3_, but also included indium metal, In_2_(SO_4_)_3_, and InCl_3_. The toxicity profiles of these compounds, though not fully elucidated, are unlikely to be the same. Our study indicates differential pulmonary toxicity between them. Of course, the different dosage for the different indium compounds may limit comparability between the four compounds. Pathobiology studies using H&E, Masson’s trichrome, and PAS staining showed obvious differences among effects of ITO/In_2_O_3_ and In_2_(SO_4_)_3_/InCl_3_ exposure. Alternatively, if intra-alveolar accumulation of surfactant and characteristic pulmonary alveolar proteinosis is triggered by a specific underlying mechanism, then perhaps only specific forms of indium could induce such reactions. The histopathological characteristics of ITO and In_2_O_3_ rat models were: (a) interstitial fibrosis and lymphocytic infiltration; (b) intraalveolar eosinophilic exudates characteristic of pulmonary alveolar proteinosis and numerous cholesterol clefts and giant cells containing brown particles; (c) focal eosinophilic exudates with immunoreactive SP-A, SP-D, KL-6, and GM-CSF; and (d) inflammatory infiltrated peribronchiolar fibrosis with immunoreactive NF-κB p65 and HO-1. However, the histopathological analysis suggested differential severity, with rats exposed to ITO exhibiting worse pulmonary alveolar proteinosis and fibrosis compared to those exposed to In_2_O_3_ at the same dosage. The In_2_(SO_4_)_3_ and InCl_3_ rat models also showed similar histological characteristics, except for the presence of pulmonary alveolar proteinosis. In the InCl_3_ rat models, the progressive destruction of lung tissue with chronic inflammation was evident. In contrast, no pulmonary alveolar proteinosis-like change was found. At the level of progressive inflammation reaction of rat lung tissue exposed to InCl_3_, both progressive destruction and alveolar protein deposition exist after ITO exposure. The results of human and animal experiments show that the inflammatory response is the main pathological pathway in the development of indium lung disease. Findings from our in vivo studies demonstrated that intra-alveolar accumulation of surfactant (immunohistochemistry) and characteristic cholesterol clefts granulomas of indium lung disease (PAS staining) were triggered by a specific form of indium (ITO or In_2_O_3_). We speculate that the cholesterol-ester, which caused the development of cholesterol granulomas, was derived from the destruction of tissues or cells of the lung. These results support the proposed diagnosis of lung injury caused by the inhalation of ITO.

Furthermore, in rats exposed to four forms of indium compounds by intratracheal instillation, the indium accumulation in rat lungs and serum increased with indium treatment dose in a dose-dependent manner. Taking into consideration the lung injuries of four different forms of indium-treated rats, it is suggested that the macrophage dysfunction of rats in pulmonary toxicity experiments may be due to pulmonary damage caused by indium exposure. Note that the actual persistence of indium in the human lung (and serum) may be different than that predicted in our rat models because the models do not account for the combined exposure outcomes of different forms of indium compounds. In this context, it is important to note that neither serum indium nor urine indium differentiates between the various indium compounds used at the plant; thus, their use must be combined with some specific serum markers. In addition, the living environment of experimental rats was relatively simple, and they were generally only exposed to indium compounds. Compared with the rats, humans are exposed to a more complex environment and may be exposed to multiple environmental factors. These risk factors have super-imposed, complex effects on the human body. Further studies should be performed to understand the mechanism of action of indium in humans.

## Conclusions

This study used population studies and animal experiments to explore the effect and mechanism of action of indium on lungs in humans and rats. We observed several associations between indium exposure and serum biomarkers of early effect in this cross-sectional epidemiological study of indium-exposed workers. Although these results require replication in other exposed populations, they provide impetus for future prospective cohort studies of indium-exposed workers to establish temporal relationships with biomarker and overt health outcomes, such as interstitial pneumonia, pulmonary alveolar proteinosis, and cancer. Consistent with our hypothesis, protein expression in rat lung tissue was quite comparable to serum biomarkers in workers, demonstrating the same health outcomes and similar relational patterns. Using the rat models, effects of the four forms of indium encountered in a workplace were evident: indium in the form of ITO would induce pulmonary alveolar proteinosis, followed by indium in the form of In_2_O_3_, however, not indium in the form of In_2_(SO_4_)_3_ or InCl_3_. We conclude that not all forms of indium will cause pulmonary damage in the same manner, which is an important consideration in indium workers for exposure assessment, screening of serum biomarkers, and epidemiological studies. In addition, the results of human studies and animal experiments show that the inflammatory response is the main pathological pathway in the development of indium lung disease. Our study provides new evidence for adverse pulmonary effects of occupational exposure to indium, demonstrating a need to regulate permissible exposure levels by health-based occupational exposure limits, and implement corresponding preventive measures in the workplaces, including a new occupational exposure limit of indium in China.

## Data Availability

The authors declare that the data supporting the findings of this study are available within the paper and its supplementary information files.
